# Koopman Mode Analysis of agent-based models of logistics processes

**DOI:** 10.1371/journal.pone.0222023

**Published:** 2019-09-11

**Authors:** James Hogg, Maria Fonoberova, Igor Mezić, Ryan Mohr

**Affiliations:** 1 Aimdyn, Inc., Santa Barbara, CA, United States of America; 2 University of California Santa Barbara, Santa Barbara, CA, United States of America; Indian Institute of Technology Madras, INDIA

## Abstract

Modern logistics processes and systems can feature extremely complicated dynamics. Agent Based Modeling is emerging as a powerful modeling tool for design, analysis and control of such logistics systems. However, the complexity of the model itself can be overwhelming and mathematical meta-modeling tools are needed that aggregate information and enable fast and accurate decision making and control system design. Here we present Koopman Mode Analysis (KMA) as such a tool. KMA uncovers exponentially growing, decaying or oscillating collective patterns in dynamical data. We apply the methodology to two problems, both of which exhibit a bifurcation in dynamical behavior, but feature very different dynamics: Medical Treatment Facility (MTF) logistics and ship fueling (SF) logistics. The MTF problem features a transition between efficient operation at low casualty rates and inefficient operation beyond a critical casualty rate, while the SF problem features a transition between short mission life at low initial fuel levels and sustained mission beyond a critical initial fuel level. Both bifurcations are detected by analyzing the spectrum of the associated Koopman operator. Mathematical analysis is provided justifying the use of the Dynamic Mode Decomposition algorithm in punctuated linear decay dynamics that is featured in the SF problem.

## Introduction

An agent-based model (ABM) is a computational technique in which behavior of individual agents is encoded by simple rules, and the outcomes are observed at the scale of the system [1]. Simple rules of behavior for individuals can lead to complex, system-scale emergent phenomena [2, 3]. ABMs are relevant to modern modeling problems because their essential feature of interacting micro-scale agents leading to macro-scale dynamics resembles many of the decentralized, highly-interacting social [4–9] and economic [10, 11] systems of today.

Beyond demonstrating the existence of emergent phenomena for their own sake, the prime importance of ABM results is their analysis for the purpose of decision making. Agent-based models have been analyzed with applications to both governmental and business decision making interests. Das and Hanaoka [12] examined an ABM of humanitarian logistics applied to the allocation of relief supplies by disparate stakeholders. Démare et al [13] studied a highly-detailed model of goods transport between a port and inland regions by different levels of agents on a dynamic graph. Binmad and Li [14] use an ABM approach to demonstrate the benefits of a forgiveness method to improve reputation systems in online commerce and social communities. Stavash et al [15] present an ABM in a war-gaming context to model the logistics of military operations.

Analysis of the results of ABM can benefit from data processing techniques that reveal details of the system behavior not readily apparent from visual examination of the model outputs, such as the nature of the time-dependent dynamics of a system and sensitivity to agent parameters. Koopman Mode Analysis (KMA) is a data processing technique that lends itself to such analyses. In this work, KMA was applied to identify transitions between different types of dynamics in numerical simulations of two different agent-based models: one simulating a large-scale, emergency medical treatment facility (MTF) system and one simulating a transport logistics situation which can considered as a simple supply chain management (SCM) problem.

Koopman Mode Analysis is a mathematical technique based on the spectral theory of the Koopman operator [16–19]. The Koopman operator gives a linear representation of a complex, nonlinear or stochastic process without any loss of information. It does this by looking at the evolution of observables on the state space of the process rather than tracking the full state directly. This is advantageous since it is often impossible, intractable, or of no interest to fully specify the state space or the process—one merely needs to look at how observables on the system evolve in time.

Having its roots in harmonic analysis, KMA decomposes an observable’s signal into components having simple temporal dynamics plus a term dealing with the so-called continuous spectrum that one can think of as the noise term. It is analogous to how a Fourier transform decomposes a signal into sinusoids with specified frequencies and phases. KMA, however, gives more. First, in addition to identifying purely oscillating components like the Fourier transform, KMA can identify growing or decaying temporal signatures (with or without oscillation). Thus KMA components have a temporal signature given by a complex exponential rather than a sinusoid. Second, KMA has a spatial component. To each temporal signature, there is identified a complex spatial structure. Thus KMA represents an observable’s signal as a linear combination of these spatial structures (called “modes”) multiplied by their temporal coefficients. An important feature of KMA is that it can be deployed to data-only systems, namely those that do not have a model associated with them. However, in this paper we apply it to outputs of two ABM systems on which detailed understanding of the impact of the underlying methodology can be discerned.

The first ABM system analyzed in this paper consists of medical facilities receiving and outputting battlefield casualties, where the input rates of casualties and the patient capacity and treatment efficacy of the medical facilities determines the dynamics of patient survival rates. The second system involves mobile fuel delivery assets (representing, e.g., ships) traversing a network of fuel-consuming sites, the dynamics of which depend on the site fuel capacity and the ability of the assets to keep sites supplied with fuel.

The two systems displayed two different kinds of dynamics, one of which was primarily stationary with fluctuations about a mean, and the other dominated by linear decay. Linear decay dynamics have not previously been treated using KMA methods, and here we show a theoretical understanding of it in terms of degeneracy of eigenvalues. From these numerical and theoretical results, we show that KMA provides an effective method to study qualitative transitions in large logistics systems.

This paper is organized as follows: In the Materials and Methods section, we first provide an overview of Koopman Mode Analysis (KMA) and the Dynamic Mode Decomposition (DMD) algorithm used to compute Koopman eigenvalues and modes, then introduce the MTF and SF logistics simulations. In the Results section we describe the results of the MTF and SF logistics simulations and the understanding of their dynamics made possible by KMA. We also provide a theoretical description of KMA applied to linear decay dynamics with random increases and show that KMA theory correctly predicts a characteristic result observed in the analysis of ABM results. The Discussion and Conclusions section discusses the significant findings of this work and presents our conclusions about these systems and the utility of KMA in describing them.

## Materials and methods

### Koopman Mode Decomposition (KMD)

Koopman Mode Analysis (KMA) decomposes a complex signal, $\left\{g_{t}\in R^{n}:t\in I\subset R\right\}$, into a linear combination of spatial structures that have simple temporal dynamics plus a “noise” term as

$$\begin{array}{c} g_{t}=\sum_{j=1}^{\infty }e^{\lambda_{j}t}v_{j}+e^{{\bar{\lambda }}_{j}t}{\bar{v}}_{j}+n_{t}, \end{array}$$ (1)

It does this by computing a data-driven spectral analysis of an underlying family of linear operators which induce the evolution of the signal. In (1), λ_*j*_ = *a*_*j*_ + *iω*_*j*_ is an eigenvalue of the underlying family of operators, a complex frequency determining the temporal signature; **v**_*j*_ is the mode which determines the spatial structure of the associated temporal signature; the overlines denote complex conjugation; and $n_{t}\in R^{n}$ is the “noise” term due to the continuous spectrum of the family of operators. We remark that the noise term does not necessarily need to have Gaussian statistics as is assumed in many other contexts.

The family of operators is induced as follows. We assume that $\left\{g_{t}\in R^{n}:t\in I\right\}$ is a stochastic process. There is an underlying state space *Z*, a flow *S*^*t*^: *Z* → *Z* (*t* ∈ *I*), and a vector-valued function $g:Z\to R^{n}$ such that

$$\begin{array}{c} g_{t}=g\circ S^{t}. \end{array}$$ (2)

The composition operation on the right-hand side induces a family of linear, infinite-dimensional operators (called the family of Koopman operators) $U^{t}:F\to F$, where $F=\{g:Z\to R^{n}\}$ is a vector space of functions, satisfying

$$\begin{array}{c} U^{t}g=g\circ S^{t} \end{array}$$ (3)

and therefore the original signal can be written in terms of the family of Koopman operators as

$$\begin{array}{c} g_{t}=U^{t}g. \end{array}$$ (4)

Since {*U*^*t*^} is a family of linear operators, we can analyze its spectrum. Assuming its a spectral family, the family can be decomposed as

$$\begin{array}{c} U^{t}g=\sum_{j}e^{\lambda_{j}t}v_{j}\left(g\right)+e^{{\bar{\lambda }}_{j}t}{\bar{v}}_{j}\left(g\right)+n_{t}\left(g\right). \end{array}$$ (5)

Using this with (4), gives (1) (where we have suppressed the dependence of the **v**_*j*_’s and **n**_*t*_ on **g**).

In KMA, the eigenvalues, $e^{\lambda_{j}t}$, and the modes, **v**_*j*_, from (4) are determined via the spectral analysis of a data-driven approximation of the family of Koopman operators *U*^*t*^.

In terms of a logistics problem such as the Medical Treatment Facility (MTF) problem, the components of **g**_*t*_ could represent the number of patients at each of the *n* medical facilities at time *t*. The state space and flow map (*Z* and *S*^*t*^, respectively) would correspond to the state space for the underlying ABM model and its simulation, respectively. A Koopman mode $v_{j}={\left(v_{j}^{\left(1\right)},\ldots ,v_{j}^{\left(n\right)}\right)}^{T}$ would determine which of medical facilities had its level of patients vary with a temporal signature partially characterized by the Koopman eigenvalue λ_*j*_ = *a*_*j*_ + *iω*_*j*_. For example, if $v_{j}^{\left(i\right)}$ was non-zero, then this would indicate that the number of patients at facility *i* is partially characterized by growth (*a*_*j*_ > 0) or decay (*a*_*j*_ < 0) with rate $e^{a_{j}}$ and oscillation with frequency *ω*_*j*_. For any fixed medical treatment facility *i*, its full temporal behavior would be given by

$$\begin{array}{c} g_{t}^{\left(i\right)}=\sum_{j=1}^{\infty }e^{\lambda_{j}t}v_{j}^{\left(i\right)}+e^{{\bar{\lambda }}_{j}t}\bar{v_{j}^{\left(i\right)}}+n_{t}^{\left(i\right)}, \end{array}$$ (6)

where $g_{t}^{\left(i\right)}$, $v_{j}^{\left(i\right)}$, and $n_{t}^{\left(i\right)}$ are the *i*^*th*^ components of **g**_*t*_, **v**_*j*_ and **n**_*t*_, respectively.

The most popular computational algorithm for computing KMA’s eigenvalues and modes is the Dynamic Mode Decomposition (DMD) algorithm which has been solidified as an important tool in the data-driven analysis of complex dynamical systems. First introduced into the Fluids community by P. Schmid in 2008 [20, 21]—although without reference to Koopman operator theory—there has been much subsequent research effort in improving the basic algorithm’s, and its variants’, correctness, stability, computational efficiency, and theoretical underpinnings. The book [22] gives a good introduction to the various methods and algorithms. In this paper, we use a variant of DMD recently introduced in Drmać et al. ([23], Algorithm 2) which includes an optional refinement procedure for the computed modes and eigenvalues as well as an explicit error term specifying the accuracy of the computed spectrum. We discuss the basic DMD algorithm here and refer the reader to [23] for details on the refinement procedure.

The DMD algorithm requires a finite sequence of snapshots of the signal, (**f**_0_, …, **f**_*m*_), where **f**_*k*_ = **g**_*k*Δ*t*_, as an input and returns the matrix **V** = (**v**_0_, …, **v**_*m*−1_) and the vector **Γ** = (*γ*_0_, …, *γ*_*m*−1_). The *i*-th column of **V** and the *i*-th element of **Γ** are approximations of some **v**_*j*_ from (1) and the associated eigenvalue $e^{\lambda_{j}\Delta t}$, respectively. Note that **f**_*k*_ = *U*^Δ*t*^ **f**_*k*−1_. The DMD algorithm first looks for a matrix $A:R^{n}\to R^{n}$ that approximates *U*^Δ*t*^ on the Krylov subspace $K=\text{span}\{f_{0},\ldots ,f_{m-1}\}\subset F$. That is, we are looking for a matrix representation **A** of $P_{K}U^{\Delta t}:K\to K$, where $P_{K}:F\to K$ is the orthogonal projection of functions onto K. The DMD algorithm then uses this matrix to approximate the Koopman modes and eigenvalues, **v**_*j*_ and $e^{\lambda_{j}\Delta t}$, respectively.

**Algorithm 1: DMD**

[**V**, **Γ**] = dmd(**F**)

**Input:** $F=\left(f_{0},\ldots ,f_{m}\right)\in R^{n\times (m+1)}$

1: **X** ← (**f**_0_, …, **f**_*m*−1_), **X**′ ← (**f**_1_, …, **f**_*m*_)

2: [**K**, Σ, **W**] = svd(**X**)

3: Determine numerical rank, *k*

4: **K**_*k*_ ← **K**(:, 0 : *k* − 1), **W**_*k*_ ← **W**_*k*_(:, 0 : *k* − 1), Σ_*k*_ ← Σ(0 : *k* − 1, 0 : *k* − 1)

5: $A\leftarrow K_{k}^{*}X^{\prime }W_{k}\Sigma_{k}^{-1}$, (*Schmid’s formula for $K_{k}^{*}U^{\Delta t}K_{k}$ in the SVD basis)*

6: [**S**_*k*_, **Γ**] = eig(**A**), (**Γ** = (**γ**_0_, …, *γ*_*k*−1_), **AS**_*k*_(:, *i*) = *γ*_*i*_*S*_*k*_(:, *i*), ‖**S**_*k*_(:, *i*)‖_2_ = 1)

7: **V** ← **K**_*k*_**S**_*k*_

**Return:** **V**, **Γ**

*Remark*. Note, we are indexing from 0 in Algorithm 1, **K**_*k*_ is an orthogonal basis for the *k*-dimensional Krylov subspace K, and **A** is the matrix representation of $P_{K}U^{\Delta t}:K\to K$ in the SVD basis **K**_*k*_. The Koopman eigenfrequency λ_*j*_ can be computed from γ_*j*_ via $\lambda_{j}=\frac{1}{\Delta t}\log\left(\gamma_{j}\right)$, where we take the principal value of the complex logarithm.

### KMA and logistics problems

In terms of the logistics problems, **f**_*k*_’s are quantities of interest during the evolution of the agent-based model of the logistics problem—e.g., the amount of fuel at each of the sites. At a very coarse-level the use of KMA/DMD for these problems is a recurrent 3-step procedure as time evolves.

1. Measure quantities of interest, **f**_*k*_, at time *t*_*k*_ (either from the simulation of an underlying ABM model or from a real system).
2. Input finite windows of snapshots (**f**_*k*−*m*_, …, **f**_*k*_) into the DMD algorithm and return Koopman modes and eigenvalues **v**_*j*_ and λ_*j*_ for that time window.
3. Use the eigenvalues and modes to interpret the overall health of the system.
4. Update simulation time and goto step 1:

Step 3 is the most involved step and is problem dependent. However, our goal is to use the Koopman spectrum to interpret the overall health of the systems under observation. In the results below, there will be a change in the spectrum as a simulation parameter is changed—the casualty source rate in the MTF problem, the maximum fuel capacity of a site in the ship refueling problem. As the eigenvalue distribution changes with changes in the parameter, the system will undergo a “bifurcation”, analogous to how the stable spiral in a Hopf bifurcation changes into an unstable spiral and a stable limit cycle appears as the eigenvalues of the fixed point cross the imaginary axis.

In the logistics problems, there do not exist obvious events to use in determining the bifurcation, such as there is with the Hopf bifurcation. There is a possibility of developing such theory for stochastic systems using an operator-theoretic version of spectral stability theory analogous to the one developed in [24]. The basic ingredients necessary for such theory have been developed for stochastic systems in [25], but are not available as full stability and bifurcation theory yet. Thus, here we take a more heuristic approach. In the results below, we will see the eigenvalues clustering around 0 (or 1) and then moving toward 1 (or 0) as the parameters are changed. While the distributions of the eigenvalues at the extremes of the parameter values is easy to see visually—and we can easily say the system behaves qualitatively differently for these parameter values—defining a unique bifurcation point is problematic. One way to automatically do this is as follows. Define a circle of radius *r*, centered at 1 in the complex plane, and define a threshold value 0 < *h* ≤ 1. For each parameter value, *θ*, compute the percentage of Koopman eigenvalues lying inside the circle around 1

$$\begin{array}{c} p\left(\theta \right)=\frac{\#\{\lambda_{j}:|1-\lambda_{j}|<r,j=0,\ldots ,m-1\}}{m} \end{array}$$ (7)

One definition of the bifurcation point is to define it as the parameter value, θ*, for which

$$\begin{array}{c} p(\theta^{*})=h. \end{array}$$ (8)

This definition, however, is not particularly useful for a stochastic system since θ* is not unique. A better way is to define two thresholds 0 < *h*_*g*_ < *h*_*r*_ ≤ 1. We then say the system is healthy (“in the green”) if

$$\begin{array}{c} p\left(\theta \right)<h_{g} \end{array}$$ (9)

and unhealthy (“in the red”) if

$$\begin{array}{c} h_{r}<p\left(\theta \right). \end{array}$$ (10)

This gives operators an automatic method of quickly interpreting the health of the system.

Of course, the usage of “healthy” and “unhealthy” above is dependent upon the system actually exhibiting undesirable behavior if the eigenvalues cluster around 1; for other systems this may be desirable behavior. Additionally, centering the *r*-circle around a different eigenvalue may make more sense for a particular system. Finally, the values of the parameters *r*, *h*_*g*_, *h*_*r*_ must be determined for each system under study according to the desired performance characteristics. These are topics that will be addressed in future research.

### Medical treatment facility logistics simulation overview

**Fig 1:**
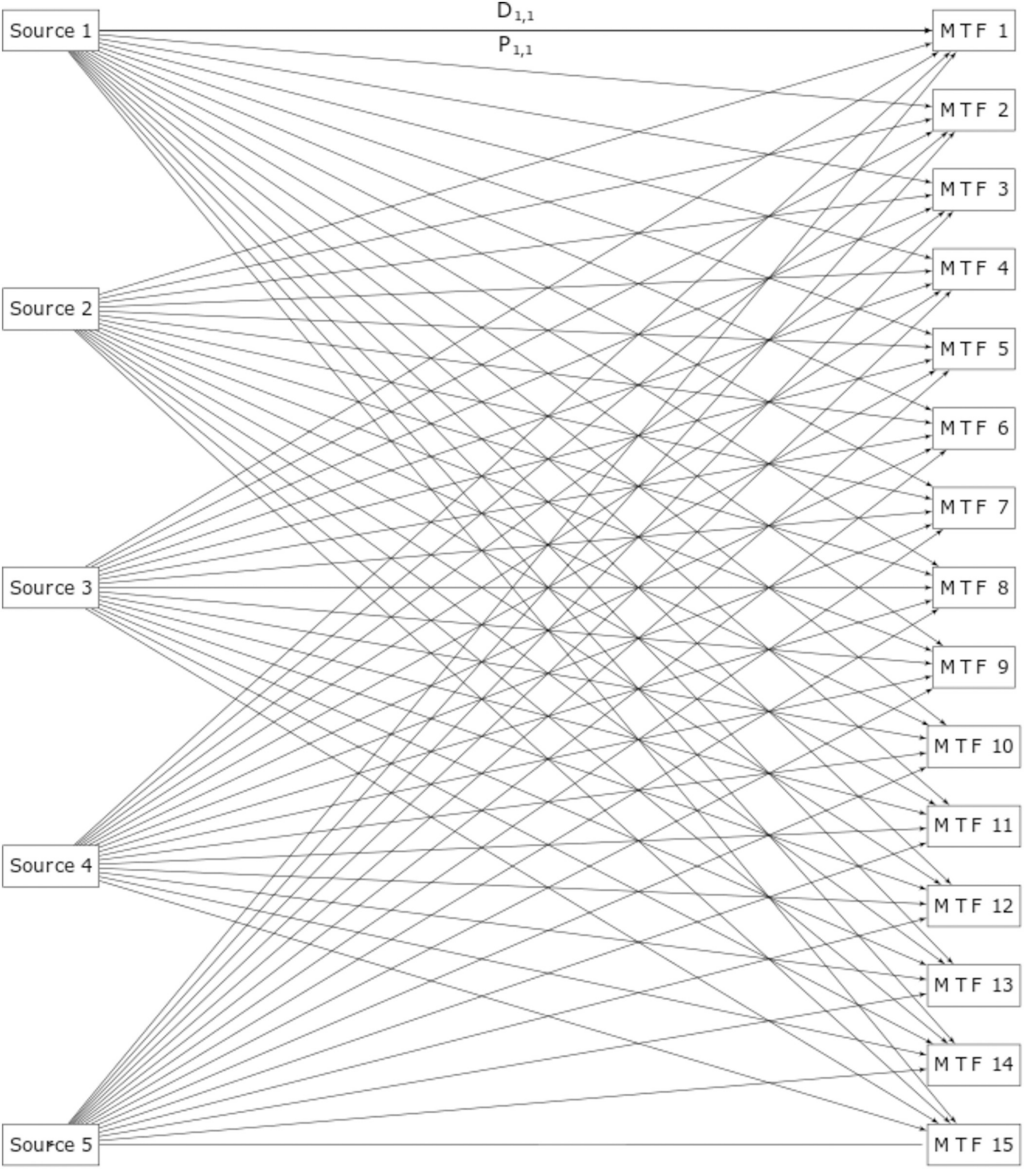
MTF network connectivity. *D*_*i*,*j*_ is the distance between Source *i* and MTF *j* and *P*_*i*,*j*_ is the probability that a given casualty generated by Source *i* will be directed to MTF *j* (for clarity only the labels between Source 1 and MTF 1 are shown).

The medical treatment facility (MTF) model consists of a network of 15 medical treatment facilities and five sources representing combat zones that produce casualties. Each time step in the simulation represents one day. Each source generates the same number *N*_*c*_ of casualties per day, where *N*_*c*_ = 30, 50, …, 330 depending on the simulation. For each MTF and source pair, there is a given probability that a casualty would be directed from that source to that MTF. The distance between each MTF and source is fixed for each pair, and for each kilometer of distance between the pair a casualty has a 1.5% chance of becoming Dead of Wounds (DOW), where each kilometer traveled is a Bernoulli trial. Each MTF has a patient capacity, where for each simulation the sum of the MTF capacities equals 800 casualties but the capacity of each MTF is randomly determined, subject to the constraint that no MTF capacity could be less than 5 casualties. Each MTF also has a fixed probability that, for each time step (day) in the simulation, a patient would become DOW or return to duty (RTD), and also an output rate, representing the number of patients (up to the occupancy of the MTF) to be transferred from the MTF to a hospital and exiting the simulation. At the end of each time step, all patients in an MTF in excess of the MTF’s capacity become DOW. Fig 1 shows the connectivity of the MTF network, where each arrow indicates a connection between each source to each MTF.

Table 1 shows the MTF to source distance for each pair and Table 2 shows the probability that a casualty from that source will be directed to a given MTF. Table 3 shows the DOW and RTD probabilities and output rates for each MTF.

**Table 1:** Distance (in km) between each source and each MTF.

	Source 1	Source 2	Source 3	Source 4	Source 5
**MTF 1**	25	41	44	37	39
**MTF 2**	31	26	28	49	32
**MTF 3**	38	41	34	31	36
**MTF 4**	44	42	33	30	47
**MTF 5**	43	30	28	29	35
**MTF 6**	45	49	37	33	34
**MTF 7**	39	49	42	33	28
**MTF 8**	39	38	49	25	46
**MTF 9**	47	48	41	42	48
**MTF 10**	31	31	27	39	30
**MTF 11**	30	35	43	28	47
**MTF 12**	29	38	48	29	46
**MTF 13**	27	26	43	46	33
**MTF 14**	26	28	33	43	34
**MTF 15**	27	38	44	30	25

**Table 2:** Probability that a casualty from a given source will be directed to a given MTF.

	Source 1	Source 2	Source 3	Source 4	Source 5
**MTF 1**	0.0064	0.0219	0.0589	0.1063	0.1054
**MTF 2**	0.1144	0.0916	0.1019	0.1062	0.0653
**MTF 3**	0.0760	0.0772	0.0273	0.1163	0.0126
**MTF 4**	0.0798	0.0609	0.0876	0.0912	0.0552
**MTF 5**	0.0177	0.1052	0.0084	0.0076	0.0148
**MTF 6**	0.0255	0.0460	0.1242	0.1078	0.0361
**MTF 7**	0.0335	0.0335	0.0722	0.0049	0.0563
**MTF 8**	0.1150	0.0962	0.0620	0.0351	0.1146
**MTF 9**	0.0638	0.0576	0.0937	0.0436	0.0289
**MTF 10**	0.1119	0.1092	0.0825	0.0218	0.0808
**MTF 11**	0.0359	0.0703	0.0598	0.0305	0.1011
**MTF 12**	0.0877	0.1057	0.1010	0.0265	0.0459
**MTF 13**	0.0964	0.0635	0.0781	0.0868	0.0453
**MTF 14**	0.0256	0.0449	0.0048	0.1038	0.1372
**MTF 15**	0.1105	0.0163	0.0376	0.1116	0.1006

**Table 3:** Characteristic parameters of each MTF. *P*_*DOW*_ is the probability that a given casualty in the MTF will die of wounds in a given time step, *P*_*RTD*_ is the probability that a given casualty in the MTF will return to duty in the given time step, and the *R*_*out*_ rate is the number of casualties (up to the total number of casualties present in the MTF) sent from the MTF to a hospital.

	*P*_*DOW*_	*P*_*RTD*_	*R*_*out*_
**MTF 1**	0.25	0.05	10
**MTF 2**	0.25	0.05	10
**MTF 3**	0.25	0.05	10
**MTF 4**	0.25	0.05	10
**MTF 5**	0.25	0.05	10
**MTF 6**	0.15	0.10	20
**MTF 7**	0.15	0.10	20
**MTF 8**	0.15	0.10	20
**MTF 9**	0.15	0.10	20
**MTF 10**	0.15	0.10	20
**MTF 11**	0.10	0.15	20
**MTF 12**	0.10	0.15	20
**MTF 13**	0.10	0.15	20
**MTF 14**	0.10	0.15	20
**MTF 15**	0.10	0.15	20

### Ship fueling logistics simulation overview

**Fig 2:**
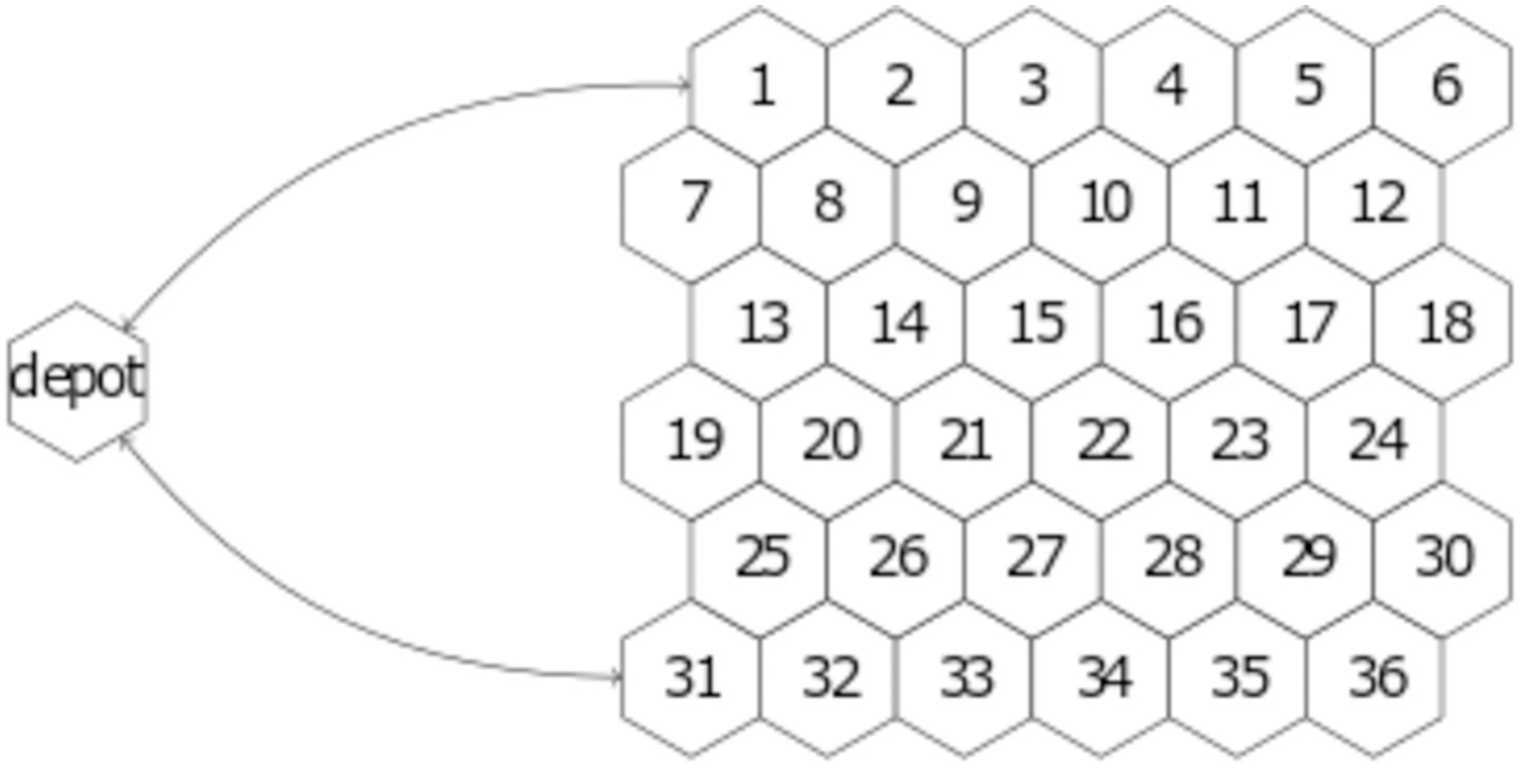
Diagram of mission site locations and depot connectivity. The sites are on a 6-by-6 hexagonal grid and sites that share an edge are connected. In addition, mission sites 1 and 31 are connected to the depot as indicated by the arrows.

The second model is an abstract representation of the logistical supply of fuel to fixed-site facilities by transport ships. The facilities are represented by a static network of 36 mission sites on a 6-by-6 hexagonal grid (see Fig 2), and the transport ships are represented by six dynamic agents referred to as assets. There is an additional site, referred to as the depot, which is distinct from the mission sites. It is connected to two of the mission sites and serves as an infinite source of fuel units.

Fuel is necessary for sites and assets to function. Each site has a single fuel level, and when the fuel level of any site reaches zero the simulation ends, with the number of time steps completed called the “mission life” for that simulation. All sites begin the simulation with an equal number of fuel units, which in this work was varied from 25 to 200 units in steps of 25. Assets have two fuel levels: 1) an internal level of the asset itself, which has a starting and maximum value of 150 fuel units and if equal to zero results in the loss of the asset and the end of its participation in the simulation, and 2) a towed fuel level, which has a starting and maximum value of 400 fuel units and represents fuel available to be transferred to a site the asset visits.

Fuel levels in sites and assets can increase or decrease from one time step to the next. Fuel levels decrease through consumption of fuel, where each site consumes one fuel unit per time step and each asset consumes two fuel units per time step. Site fuel levels increase when a visiting asset transfers its towed fuel to the site and asset fuel levels increase when the asset visits a depot as described below.

The assets all begin the simulation in the depot. In each time step of the simulation, each asset performs one action. If the asset is in a depot and its internal fuel level is less than its maximum value, then the only possible action for the asset is to remain at the depot and increase its internal fuel level at a rate of two fuel units per time step. The asset’s towed fuel value is set to its maximum value after the first time step the asset spends in the depot. If the asset is in a depot and its internal fuel level is at its maximum value, then the asset moves to a randomly determined neighboring site (site 1 or 31). If the asset is at a site, then the asset can perform one of up to three types of actions: 1) transfer some number of fuel units to the current site, where the number of fuel units transferred cannot exceed either the asset’s current towed fuel value or be such that the site would exceed its maximum fuel value, 2) move from the current site to a neighboring site, or 3) stay at the current site and do not transfer any fuel. The asset randomly chooses one action from a list of possible actions formed by all legal varieties of the three action types, i.e., the asset has an equal probability of staying at the current site, transferring one unit of fuel, transferring two units of fuel, moving to one neighboring site, or moving to another neighboring site, assuming each action was legal for the particular circumstances. Because the scale of fuel values used in the simulations was in the tens or hundreds and the number of neighbors for each site was six or less, the expected asset behavior was to stay at a site transferring fuel for each time step until the site was near its fuel capacity.

## Results

### Koopman Mode Analysis of MTF simulation results

**Fig 3:**
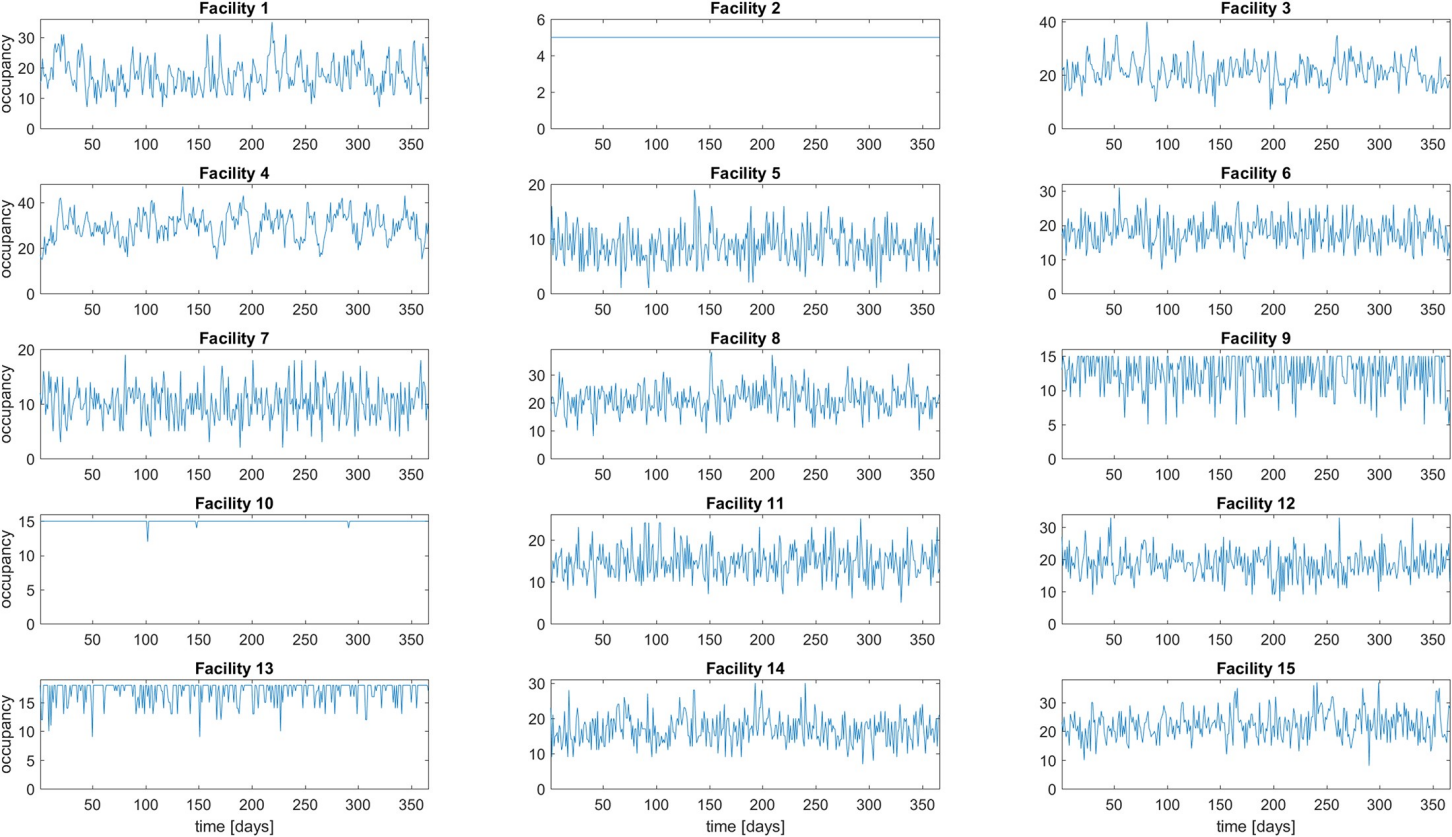
Example MTF occupancy time series, for the 90 casualties / source / day case. The horizontal axis is days and the vertical axis is MTF occupancy. The vertical axis limit of each plot is one patient more than the capacity of that MTF, therefore the fullness at each time step is approximately the ratio of the plotted occupancy to the vertical limit of the plot. E.g., MTFs 2 and 10 have fullness values equal to or close to 1 for most of the simulated period, MTFs 9 and 13 often reach a fullness of 1, and the remaining MTFs have fullness values that varying around roughly 0.5.

For each value of *N*_*c*_ (the casualty source rate), one hundred simulations were run, each with random MTF capacities subject to the constraints described in the previous section. Each simulation was run for 365 time steps (i.e. one year of simulated time). The observable value used as the KMD input was the MTF “fullness,” defined as the ratio of the occupancy of each MTF to its capacity (see Fig 3 for example time series). The KMD algorithm used was the DMD_RRR method [23].

**Fig 4:**
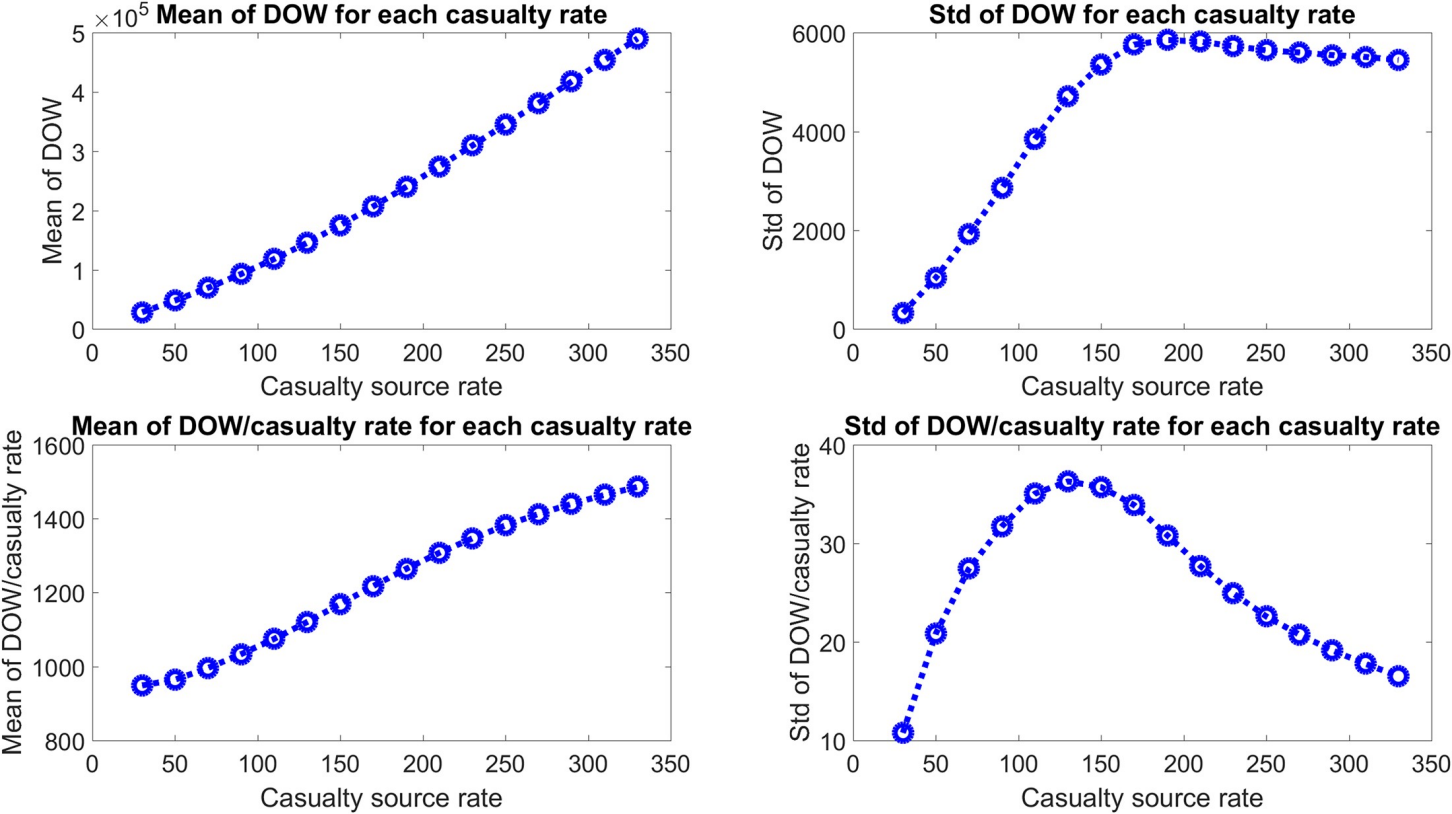
Mean and standard deviation of total Dead Of Wounds (DOW) for each casualty source rate. The top row shows the mean and standard deviation of DOW, and shows that the mean DOW increases superlinearly with casualty source rate, while the standard deviation reaches a maximum and levels out around a casualty source rate of around 175. To show the deviation from a linear increase, the bottom row shows the mean and standard deviation divided by the casualty source rate. This shows a sigmoidal shape to the mean plot and a clear peak to the standard deviation plot at around 130 casualties/source/day.

The output quantity of interest was the total Dead Of Wounds (DOW) for each simulation, where, clearly, a lower DOW was better. Fig 4 shows the mean and standard deviation for the 100 DOW values from each simulation for each casualty source rate case. The increase of the mean of DOW with casualty source rate is expected, as any MTF occupancy above the site’s capacity is converted to DOW at the end of each day, so a larger casualty source rate naturally leads to a larger DOW. It is therefore interesting to instead consider a normalized DOW, defined as the mean DOW divided by the casualty source rate, which shows non-monotonic behavior. The standard deviation of the mean DOW shows the consistency of the resulting DOW for each simulation at a given casualty source rate, which is seen to approximately plateau at a high enough casualty source rate. In contrast, the standard deviation divided by the casualty rate shows non-monotonic behavior, as it has a peak around 130 casualties/source/day, suggesting that the DOW value is most dependent on the relative MTF capacities at that casualty source rate and that MTF capacities need to be carefully determined to minimize the DOW value.

To gain greater insight into the change in system dynamics as the casualty source rate increases, it is instructive to apply Koopman mode decomposition of the fullness observable and examine the Koopman eigenvalue distributions.

**Fig 5:**
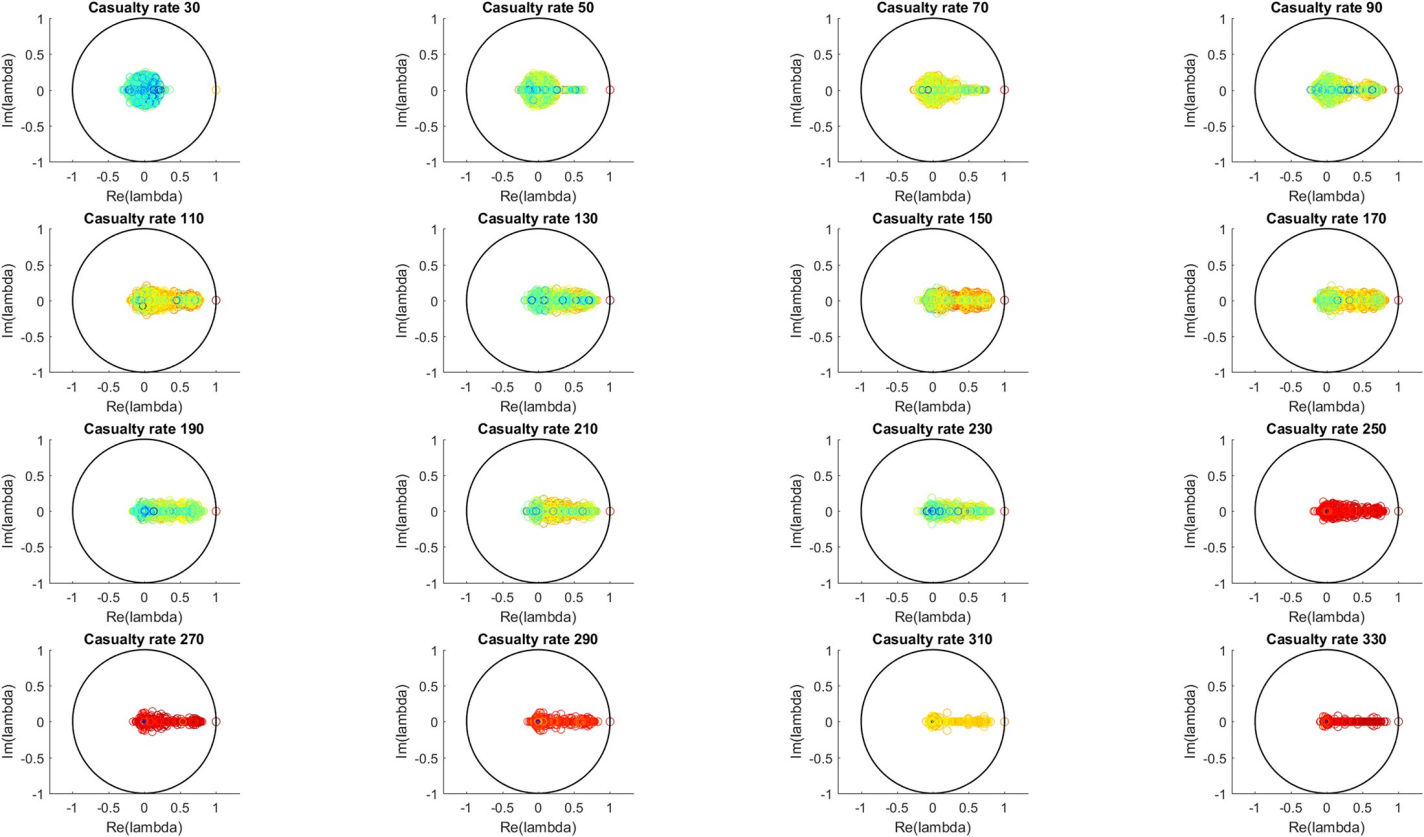
All eigenvalues for all simulations for each casualty source rate. The color scale (blue to red) indicates the L-2 norm of each eigenvalue’s associated Koopman mode. The transition from damped behavior at low casualty source rates to less damped behavior at higher casualty source rates is apparent in the shift in the eigenvalue distribution from clustering around the origin to collapsing onto the real axis and approaching the unit circle.

Fig 5 shows, for each casualty source rate, the distribution all of the Koopman eigenvalues from all 100 simulations at that casualty source rate. The eigenvalues show the expected behavior based on inspection of the MTF fullness time series. At low casualty source rate values, few or none of the MTF are near capacity so the time series show primarily damped oscillation around an equilibrium, which is reflected in the eigenvalue distribution by clustering around the origin. As the number of casualties from the sources increases, the MTF fullness values approach one and the damping effect decreases. Dynamically, this is a transition between a fluctuation-dissipation regime of fast decaying oscillations and a regime of slowly decaying or non-decaying fluctuations. As seen in the eigenvalue distributions, for increasing casualty source rate value, the imaginary component of the eigenvalues (corresponding to oscillatory behavior) tends toward the real axis, and the real component of the eigenvalues (corresponding to growth/decay behavior, and here showing the decrease in damping) tends toward more positive values.

**Fig 6:**
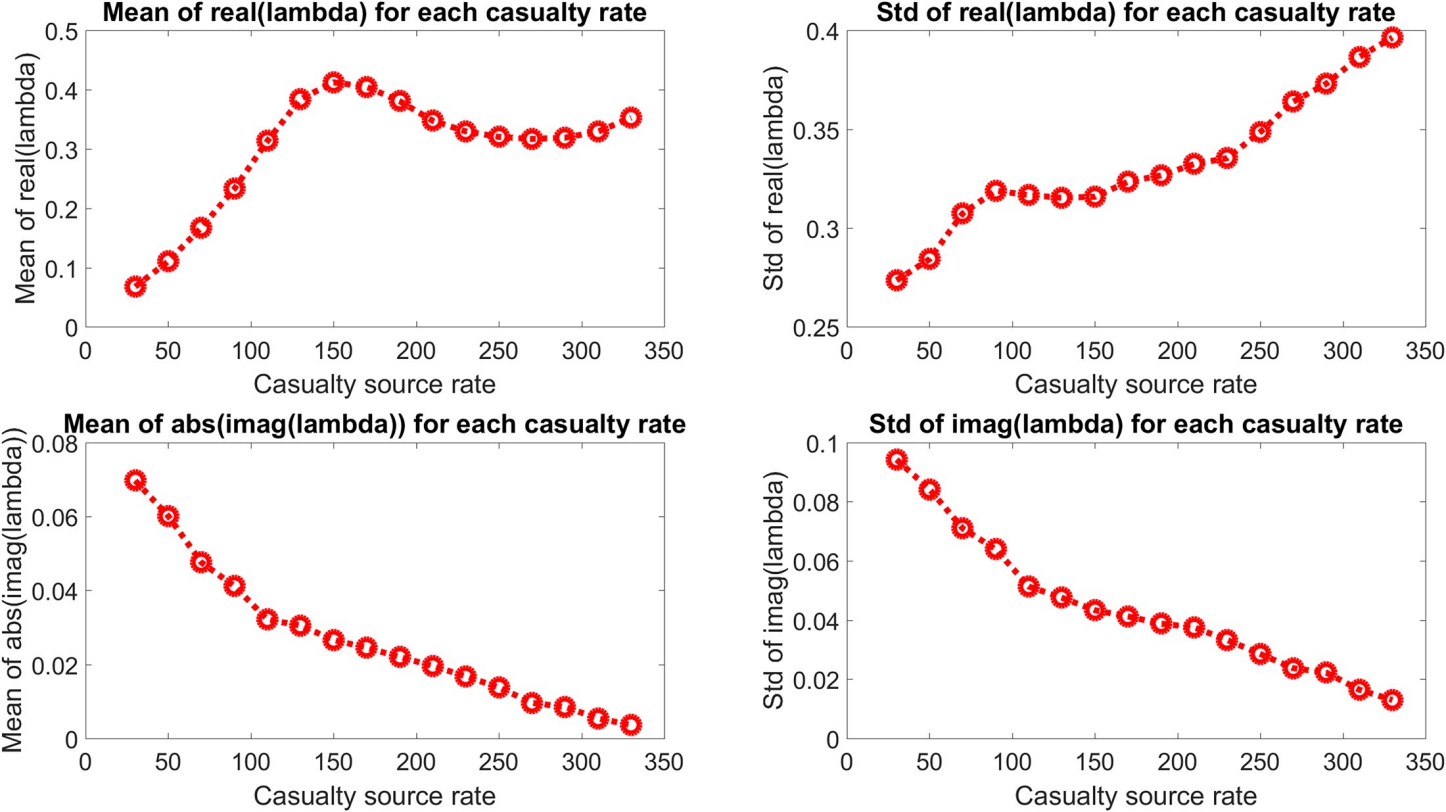
Mean and standard deviation of the real and imaginary components of the eigenvalues in Fig 5. The real components are seen to increase with casualty source rate and approach an approximate maximum, and the imaginary components are seen to approach the real axis.

The eigenvalue behavior is presented in a different form in Fig 6, which shows the mean and standard deviation of the real and imaginary components of the eigenvalues shown in Fig 5 and which show quantitatively the change in system dynamics described above. The mean real component is seen to increase to an approximately constant value with increasing casualty source rate, with a generally increasing standard deviation as well. The mean of the absolute value of the imaginary component and the standard deviation of the imaginary component are both seen to decrease with increasing casualty source rate.

Instead of considering all eigenvalues for each simulation, specific eigenvalues from each simulation and their corresponding modes can be selected. Two different eigenvalues/modes of interest are defined: the “dominant” mode and the “second” mode. These modes are defined as those whose corresponding eigenvalues have, respectively, the largest and second largest real components in each simulation.

The specific rational for the definitions of the dominant and second modes is that the long-time behavior of the site dynamics will by definition be dominated by those modes with the slowest decay rate (or equivalently, the fastest growth rate), as the time dependent coefficient of such modes in the Koopman decomposition will be larger than modes with smaller real components, over sufficiently long time scales. The mode defined as the dominant mode here will generally be the mode that is approximately equal to the mean of the data observables, and thus of limited dynamical interest. The mode defined as the second mode will better represent the mean-subtracted behavior of the system, as it includes a greater contribution of the damping effect on the system and oscillatory behavior. One could define further “higher order” modes using this approach, but the dynamical significance of such modes are less important on longer time scales, unless the real components of the associated eigenvalues are very similar to that of the second mode’s eigenvalue, because of the exponential time dependence of the mode coefficients.

**Fig 7:**
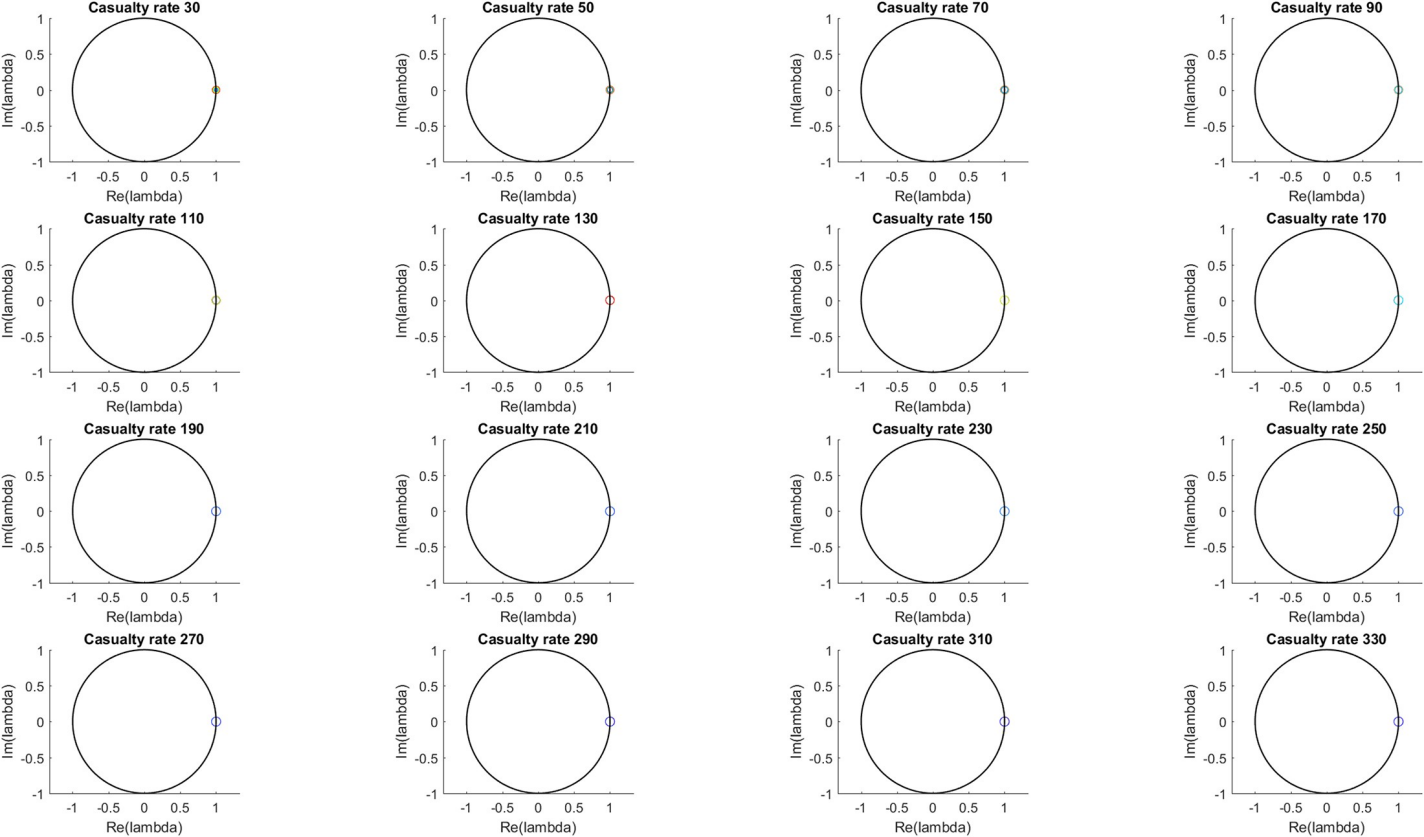
Eigenvalue distribution of dominant mode (i.e. mode with largest real eigenvalue) for each casualty source rate. The color scale (blue to red) indicates the L-2 norm of each eigenvalue’s associated Koopman mode. The dominant mode eigenvalues are seen to not vary significantly with casualty source rate, consistent with their identification as the mean patient value over the MTFs.

**Fig 8:**
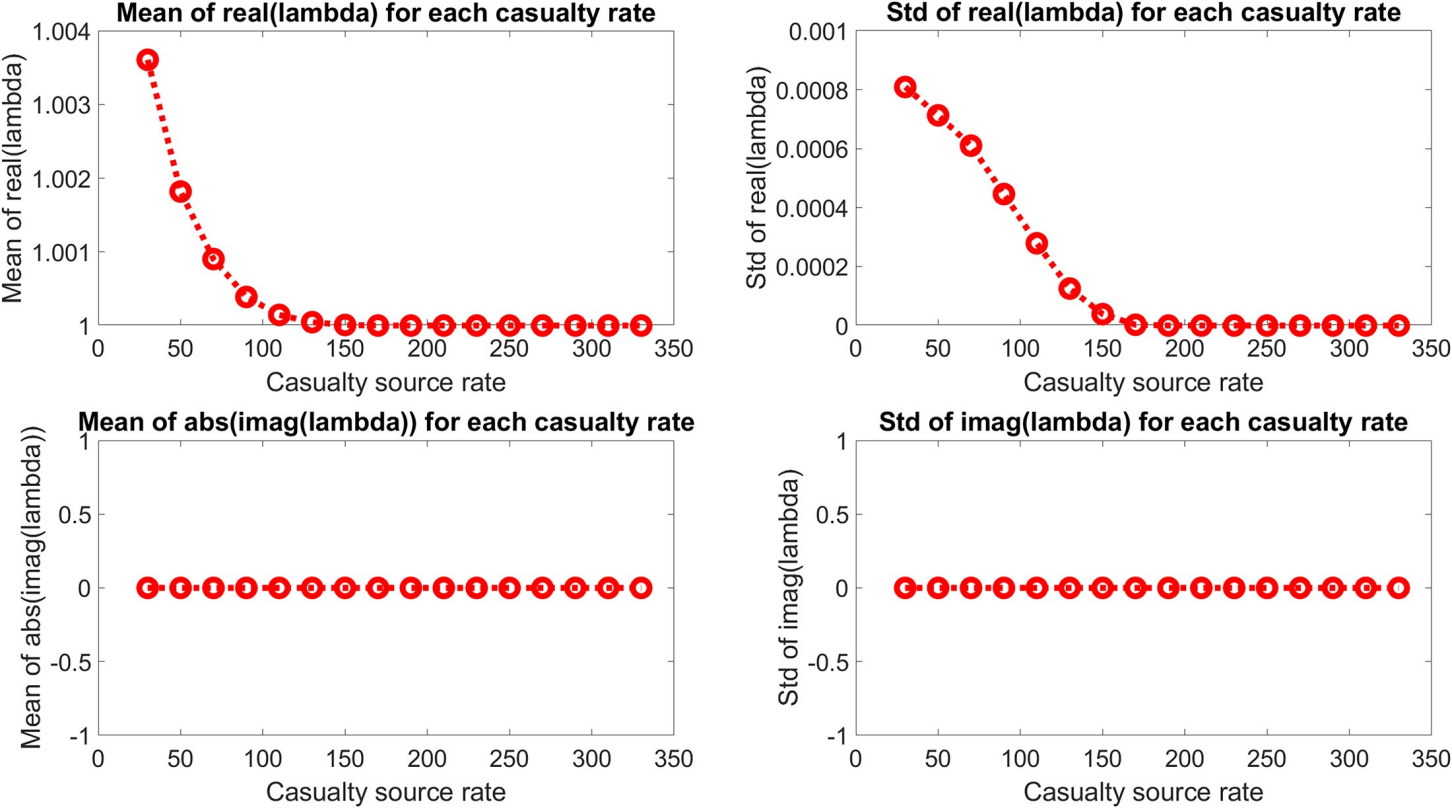
Mean and standard deviation of eigenvalue distribution of dominant mode for each casualty source rate. The dominant mode eigenvalues are seen to not varying significantly with casualty source rate.

The dominant mode is generally expected to correspond to the mean occupancy of the MTFs, in which case it will be non-oscillatory and neither growing nor decaying. Figs 7 and 8 show the eigenvalues and their statistics of the dominant mode, which is indeed consistently at or near (1,0) and does not vary meaningfully based on the casualty source rate.

**Fig 9:**
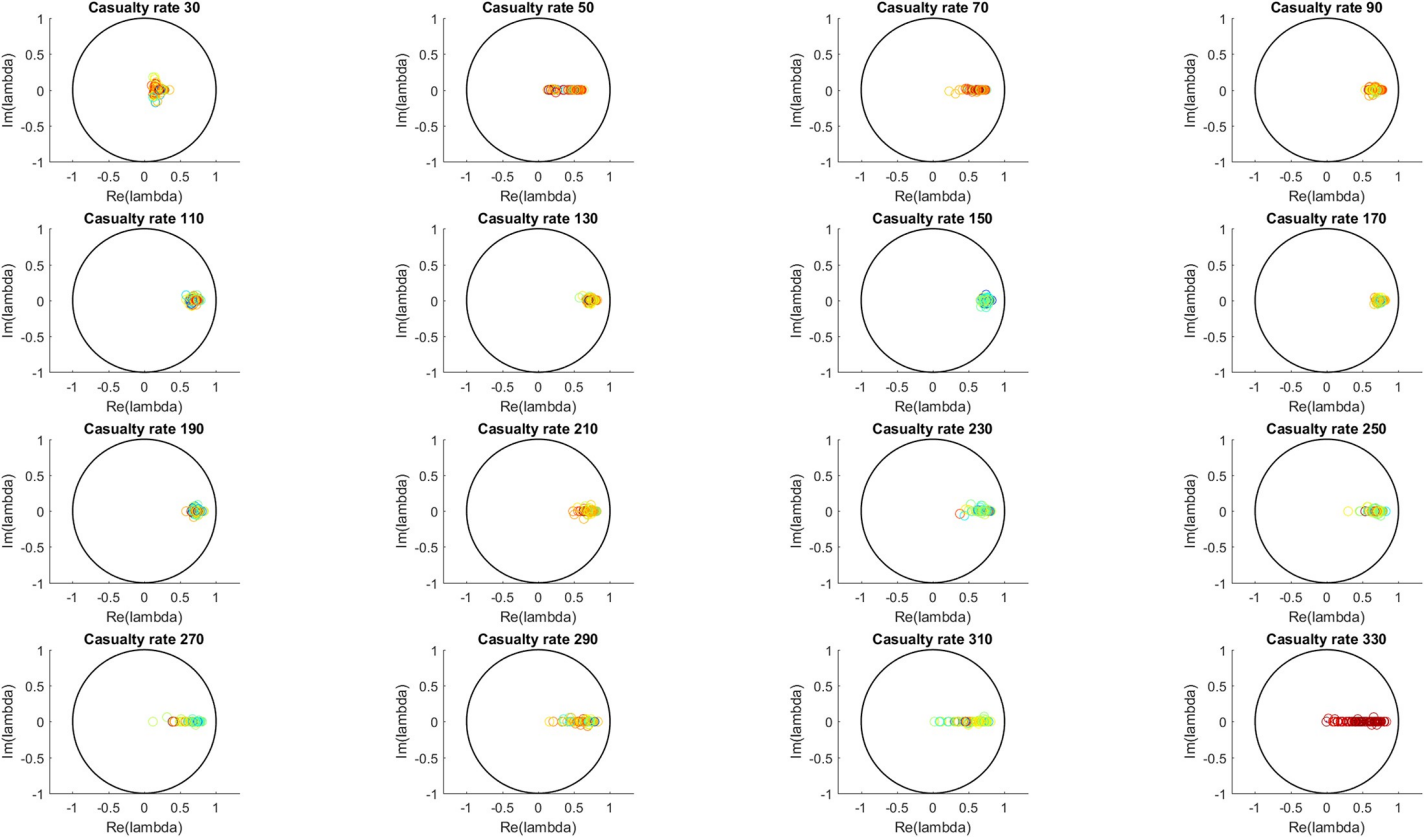
Eigenvalue distribution of second mode for each casualty source rate. The color scale (blue to red) indicates the L-2 norm of each eigenvalue’s associated Koopman mode. The second mode eigenvalues are seen to represent the general dependence on casualty source rate of the all eigenvalue case shown in Fig 5.

**Fig 10:**
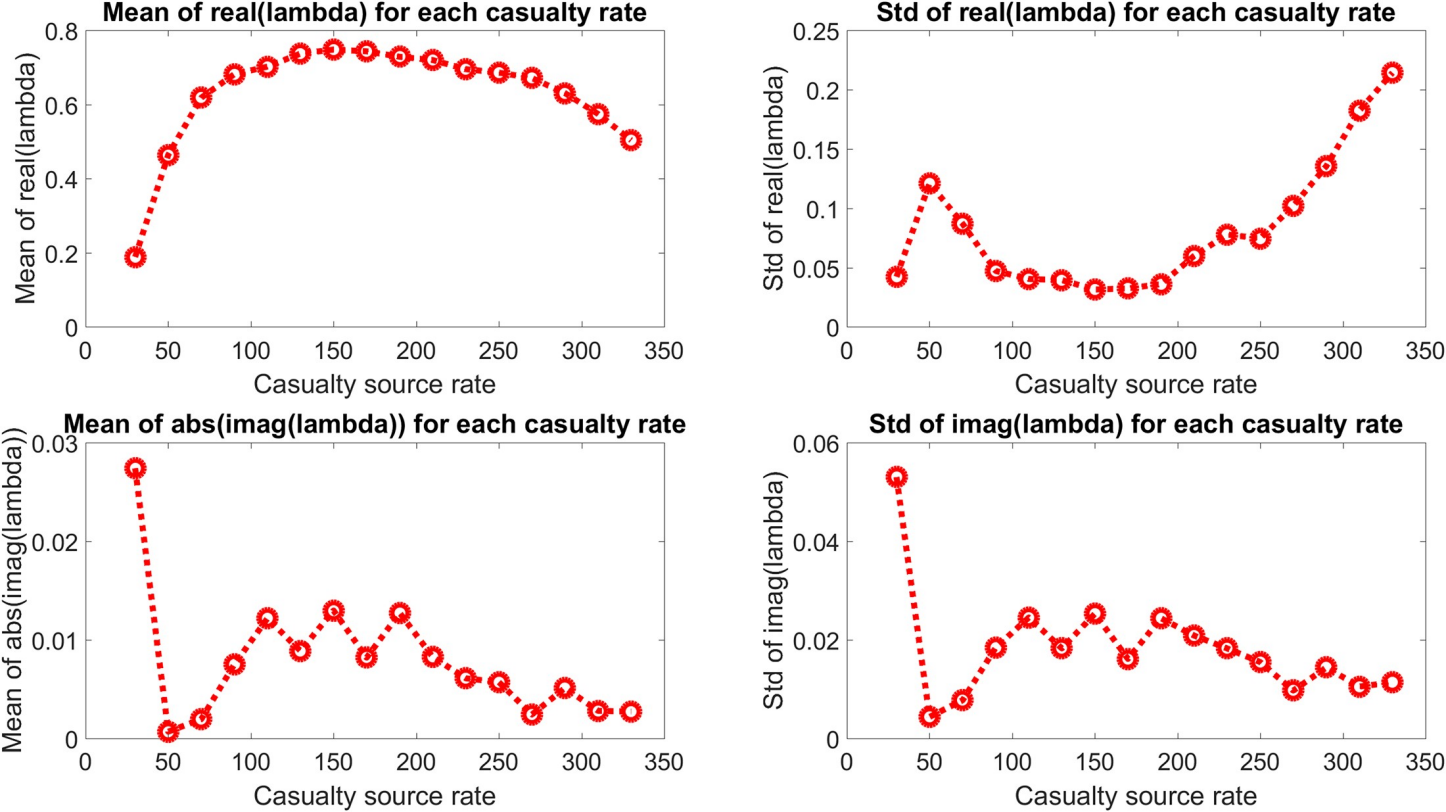
Mean and standard deviation of eigenvalue distribution of second mode for each casualty source rate. As in the all-eigenvalue case, the real component approaches the unit circle as the imaginary component approaches the real axis.

Analysis of the second mode is more useful to identify the qualitative change in behavior with increasing casualty rate. Figs 9 and 10 show the eigenvalues of the second mode and their statistics. A transition between the low casualty source rate and the high casualty source rate is apparent between casualty source rates of 30 to 110, corresponding to the transition in normalized DOW seen previously in Fig 4. This appears as the second mode eigenvalues transition from strongly damped oscillatory behavior to non-decaying fluctuations. Also the standard deviations of both the real and imaginary components decrease with increasing casualty source rate, corresponding to a transition to more similar behavior between random realizations with varied MTF capacities when the MTFs are consistently at or near capacity.

**Fig 11:**
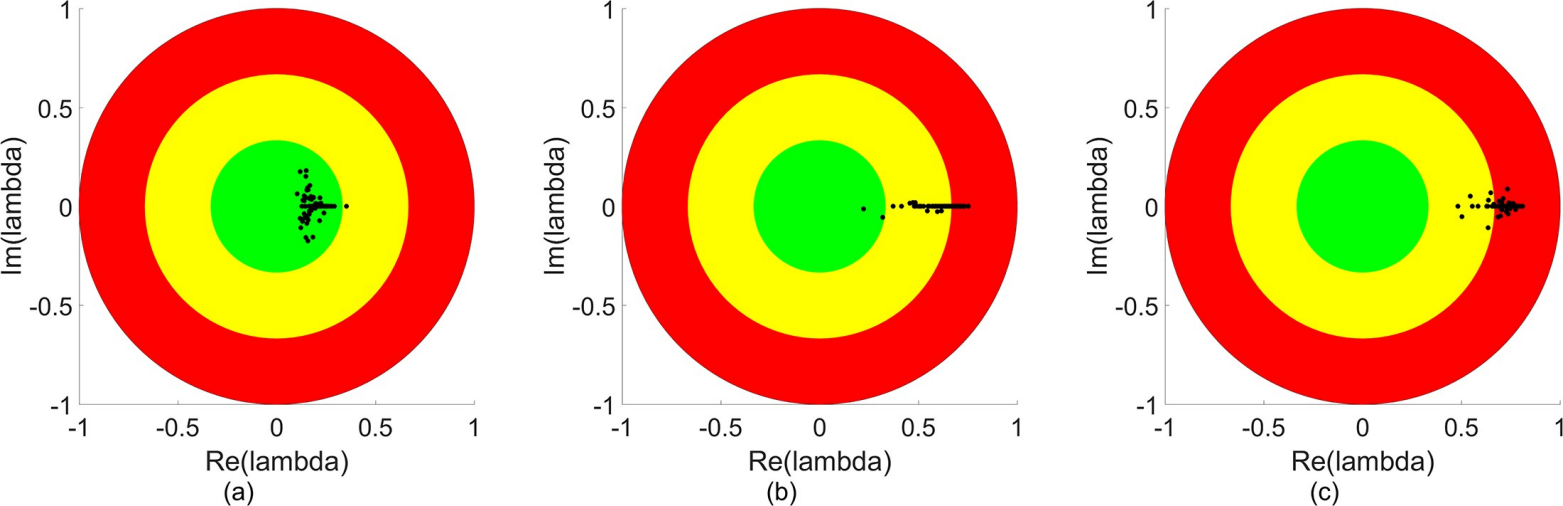
Schematic illustration of the possible use of the Koopman eigenvalue distribution as a diagnostic tool of the “health” of a dynamical system, where as noted in the text the specific radii of the colored regions shown here are arbitrary. For the case where the eigenvalue distribution is clustered in the green region (near the origin), the system is in a highly damped, fluctuation-dissipation regime and is thus likely “safe” and unlikely to exhibit extreme behavior. For the case where a significant number of eigenvalues are in the yellow region, the system damping is decreasing and the system may be approach a transition to more extreme behavior. When the eigenvalue distribution is heavily in the red region, the system is expected to be displaying fluctuations with limited or no damping and therefore extreme and potentially dangerous or damaging behavior.

The power of KMD analysis in this application is thus shown by the relation between the transition in the normalized DOW data and the behavior of the Koopman eigenvalues, particularly the second mode eigenvalue. For the general MTF case with arbitrary parameter values, the Koopman eigenvalues in general and the second mode eigenvalues in particular can be used as a diagnostic “green light, yellow light, red light” test for the state of the system. If the eigenvalues are near the origin, then the system is likely in the unsaturated, damped oscillation, low DOW state and the system is behaving as desired (“green light”). If the eigenvalues are in the intermediate region between the origin and (1,0), then the system is likely in a transition state between the low and high DOW states and potential problems may appear (“yellow light”). And if the eigenvalues are at or near (1,0), then the system is likely in the undesired, non-decaying fluctuation, high DOW state (“red light”). Fig 11 shows a schematic demonstration of this concept for second mode eigenvalues. In a simulation case, this information could be used to end suboptimal simulations to saving computing time. In a real-world MTF or other facility case, this information could be useful for decision makers to reallocate resources or take other corrective actions to minimize deaths or other loses. Fig 11 shows a schematic illustration of this concept, where the boundaries between the colored regions are chosen arbitrarily. For a specific application the boundaries of the regions would be determined based on prior data and/or theoretical considerations. By way of example, in Fig 9, one might reasonably categorize the casualty rate 30 case as being in the green light regime, as the eigenvalues remain near the origin indicating a strong damping effect; casualty rates 50 and 70 as in the yellow light regime, as the eigenvalue distribution has moved away from the origin; and higher casualty rate cases as being in the red light regime, as most or all of the second mode eigenvalues are near to unique circle, indicated weakened damping.

### Koopman Mode Analysis of ship fueling logistics simulation results

**Fig 12:**
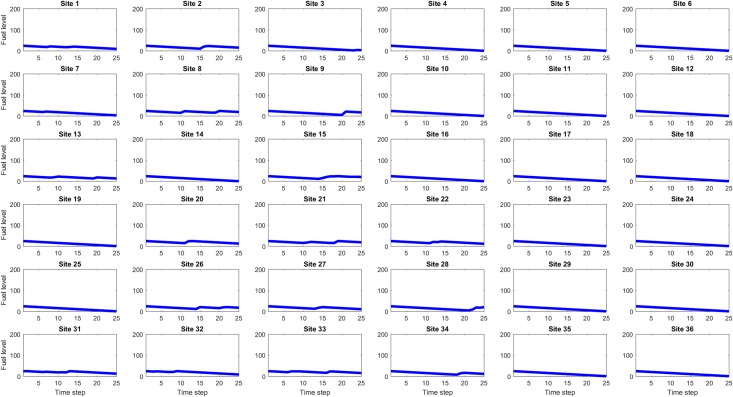
Time series of site fuel unit values, for 25 initial (and maximum) fuel units case. The linear decrease in fuel level is due to the consumption by each site of one fuel unit per time step in the simulation. The increases occasional increases in fuel level at some sites are due to refueling by mobile assets.

**Fig 13:**
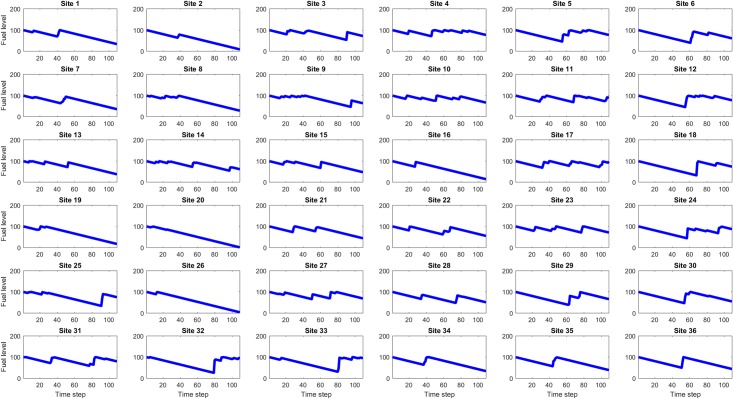
Time series of site fuel unit values, for 100 initial (and maximum) fuel units case. The longer simulation run times made possible by the higher initial site fuel value gives the assets more time to refuel sites.

**Fig 14:**
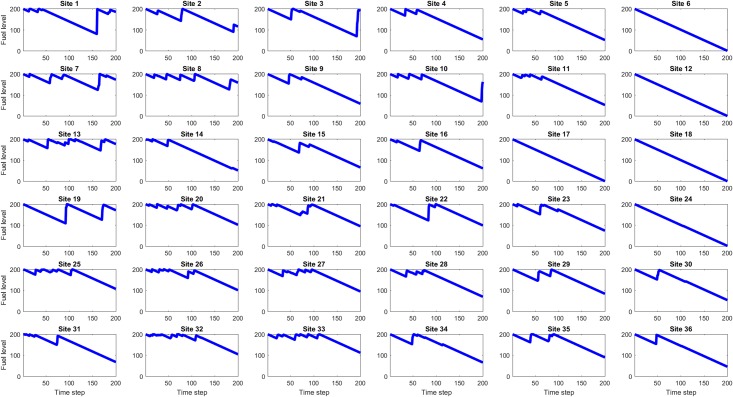
Time series of site fuel unit values, for 200 initial (and maximum) fuel units case. The simulation run times in this case are approximately twice as long as the 100 fuel unit case and consequently the assets have even more time to refuel sites, however it is seen that some site, such as 6, 12, 18, and 24, are still seldom or never refueled.

For each of the eight values of site maximum fuel capacity (25, 50, … 200), one hundred simulations were run and for each simulation the site fuel values at each time step were the observables used for Koopman Mode Analysis using a DMD-based algorithm (DMD_RRR). Figs 12, 13 and 14 show example site fuel value time series for single simulations with maximum site fuel values of 25, 100, and 200 fuel units. It is seen that the larger maximum site fuel values enable longer mission life times and more opportunities for assets to refuel sites, thus leading to more complicated dynamical behavior.

**Fig 15:**
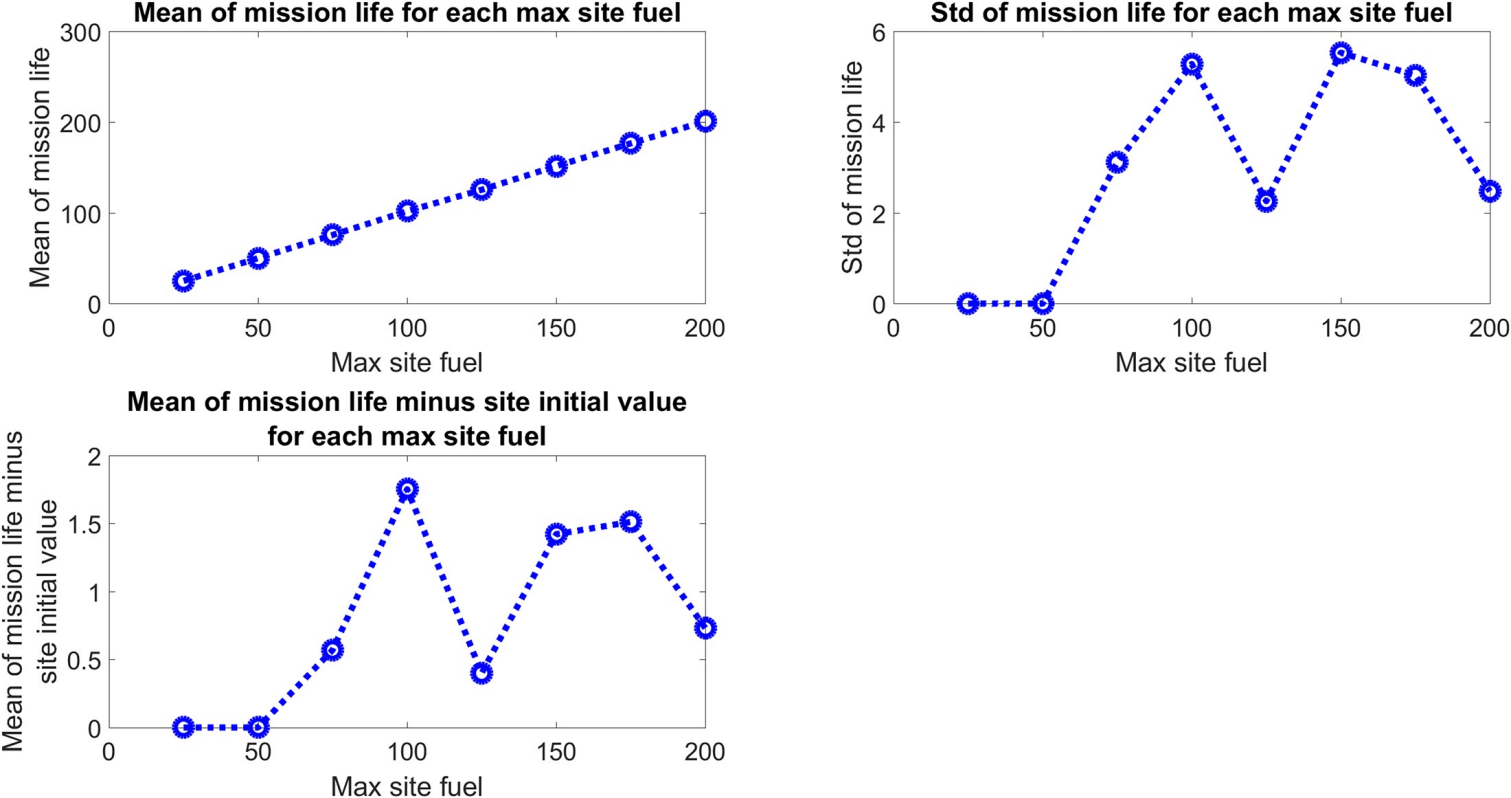
Mission life statistics. The mean mission life increases with the maximum and initial site fuel value, and the standard deviation of mission lives also generally increases with maximum and initial site fuel value. To show the deviation from a purely linear increase in mission life, the bottom left plot shows the mean mission life with the minimum possible mission life subtracted off. This result shows the mission-life extending effect due to refueling by the assets.

The mission life for each simulation was also recorded and processed. Fig 15 shows the mean and standard deviation of the mission life of each of the 100 simulations for each initial fuel value. Also shown is the difference between the mean mission life and the initial fuel value for each site, to better show the deviation of the mean mission life from a linear increase. The simulation ends when any site reaches zero fuel value and so the initial fuel value is the minimum possible mission life. This also explains the approximately linear increase in mean mission life times for each maximum site fuel value, as any site not refueled by an asset within the minimum possible mission life will cause the simulation to end. The standard deviation of mission life times for each maximum site fuel value is also consistent with the increased possibility of the assets refueling all sites at least once, leading to mission life times longer than the minimum value. The deviation of the mean mission life values from the linear, minimum mission life values shows the same behavior as the mission life standard deviation, where larger maximum site fuel values increase the likelihood of all sites being refueled at least once before reaching zero fuel value.

Koopman Mode Analysis (KMA) processing was performed on the ship fueling logistics simulations to study the change in dynamical behavior as the maximum site fuel value was increased. A DMD-based algorithm for Koopman Mode Decomposition (KMD) was applied to the site fuel value time series from each of the 100 simulations for each maximum site fuel value, i.e. for each of the eight maximum site fuel values, KMD was performed on each of the 100 simulations, for a total of 800 applications of KMD. For each simulation, the KMD input consisted of a 2-D matrix with 36 columns and a number of rows each to the mission life in number of time steps. Each row consists of the fuel values of each of the 36 sites during a particular time step. The outputs of the KMD for each simulation are an equal number of Koopman modes and eigenvalues, where the mode is a 36 component vector and each eigenvalue is a single complex number that determines the time-dependence of the mode, specifically its real part determines the exponential growth/decay behavior and the imaginary component determines the oscillatory behavior of the mode. Examination of the magnitude of the elements of each mode gives insight into the behavior of each mode, as each element in a mode vector corresponds to a single site and so an element having large magnitude indicates that the corresponding site’s dynamical behavior can be described, at least in part, by that mode’s eigenvalue.

The analysis consisted of examination of the distributions of three sets of eigenvalues: 1) all of the eigenvalues from each simulation for each maximum site fuel value, 2) a single eigenvalue corresponding to the so-called “dominant” mode from each simulation for each maximum site fuel value (i.e. 100 eigenvalues per maximum site fuel value), and 3) a single eigenvalue corresponding to the so-called “second” mode. As for the MTF case, the dominant and second modes were defined, respectively, as the mode with the largest real eigenvalue and the mode with the second largest real eigenvalue, where these definitions were chosen to capture the dominant long-time behavior of the site dynamics.

**Fig 16:**
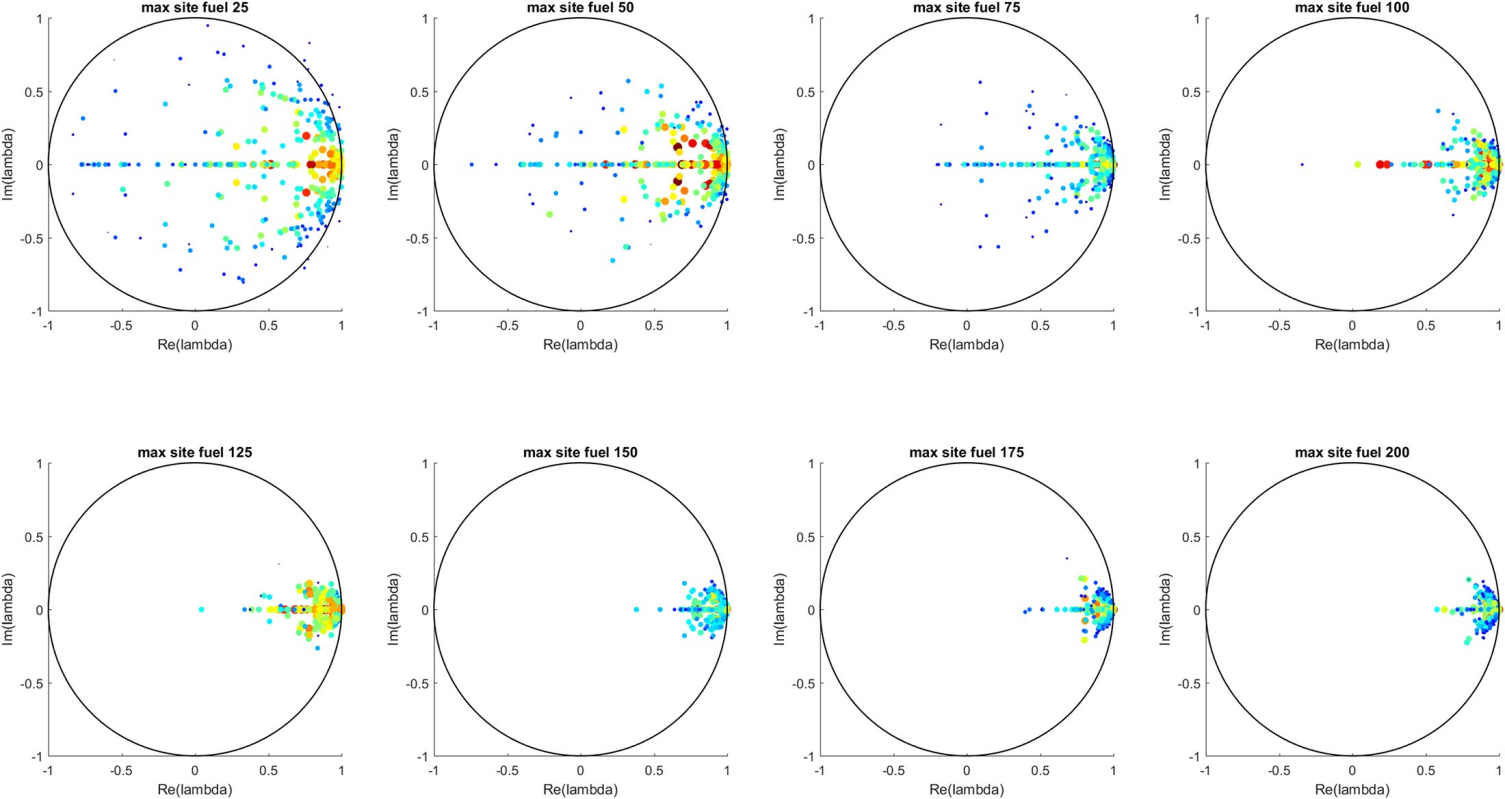
All eigenvalues for each maximum site fuel value. The eigenvalue color scale (blue to red) shows the L-2 norm of each eigenvalue’s associated Koopman mode. Similar to the MTF case, the eigenvalues are seem to cluster toward the real axis and unit circle with increasing maximum site fuel values.

**Fig 17:**
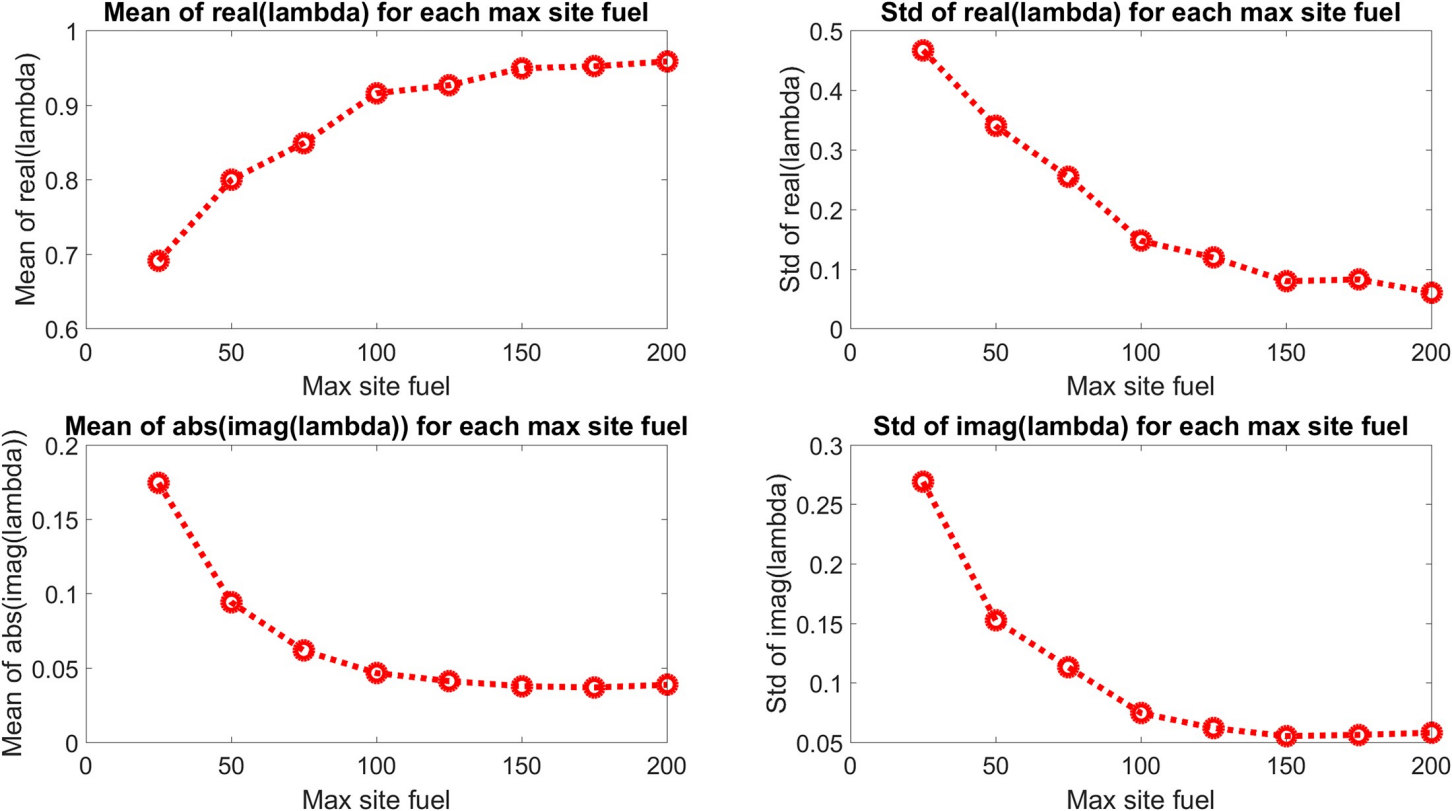
Mean and standard deviation of all eigenvalues for each maximum site fuel value. The real component is seen to go towards the unit circle and the imaginary component goes toward the real axis with increasing maximum site fuel values.

Fig 16 shows all the Koopman eigenvalues for each maximum site fuel value and Fig 17 shows the mean and standard deviation of the real and imaginary components of each eigenvalue. These results show a transition from more strongly damped behavior at lower values of maximum site fuel value to less strongly damped behavior at higher values of maximum site fuel values.

**Fig 18:**
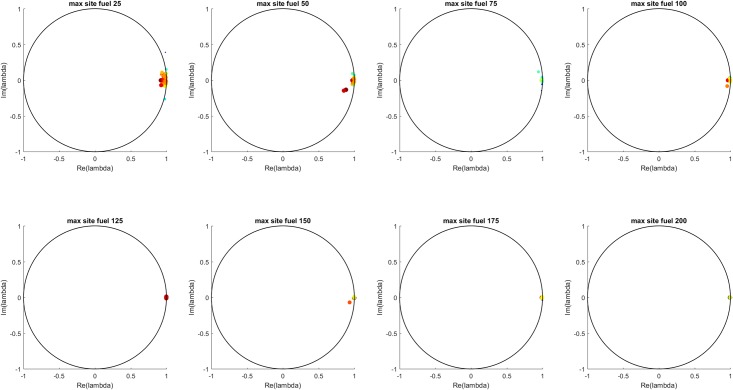
Eigenvalues of the dominant mode for each simulation for each initial fuel value. The eigenvalue color scale (blue to red) shows the L-2 norm of each eigenvalue’s associated Koopman mode. The dominant mode eigenvalues mostly represent the mean of the site fuel values and do not depend strongly on maximum site fuel value.

**Fig 19:**
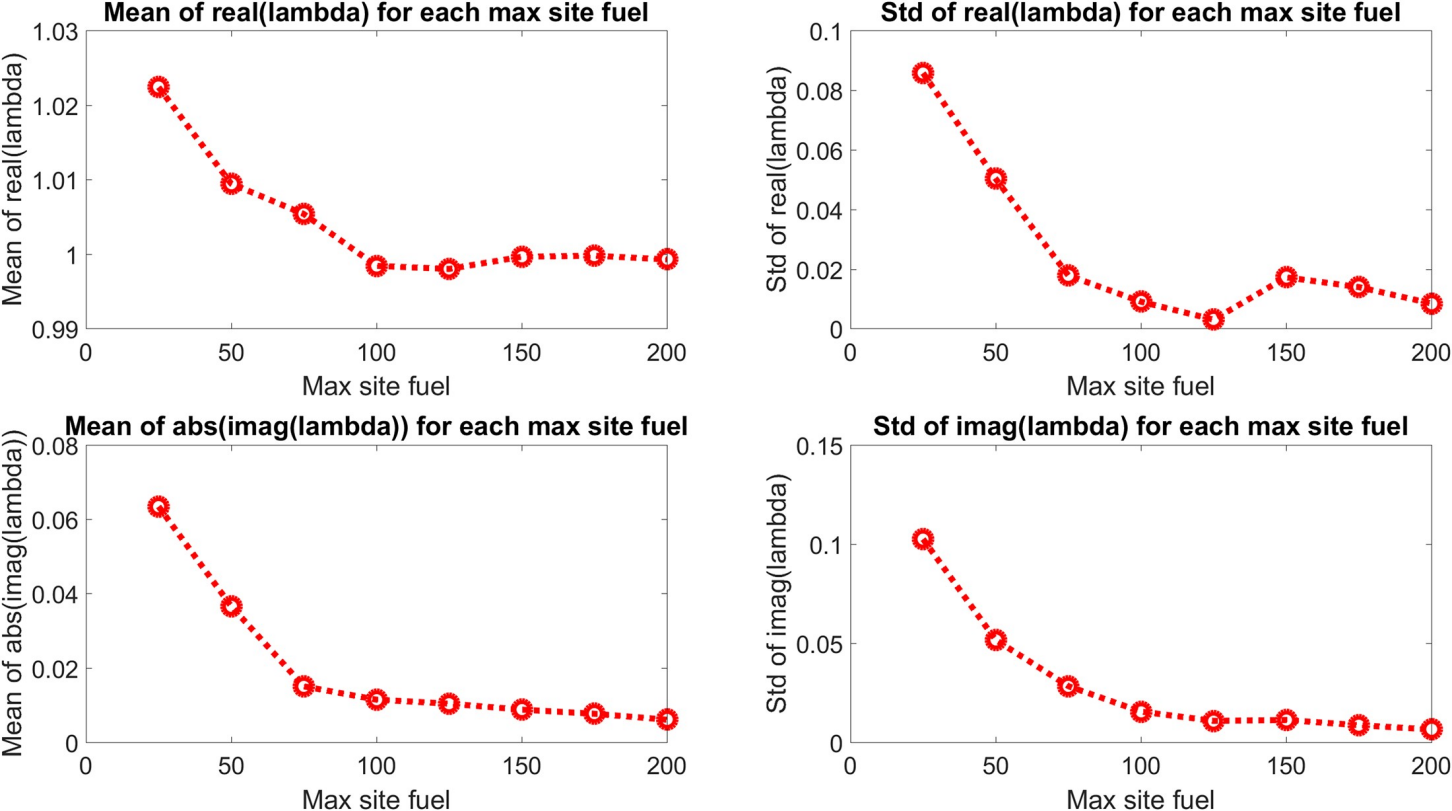
Mean and standard deviation of dominant mode eigenvalue components. The eigenvalues do not show a strong dependence on maximum site fuel value.

**Fig 20:**
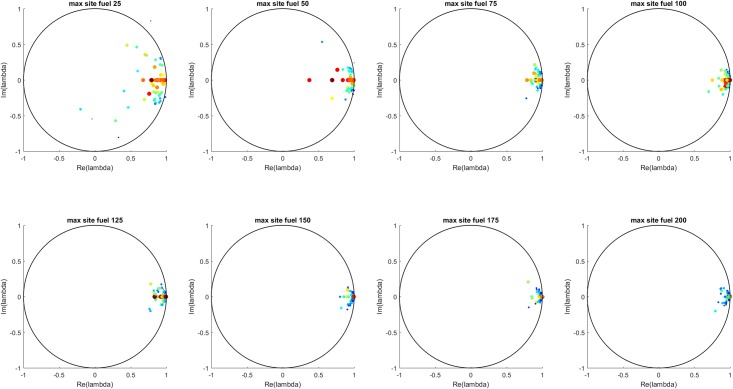
Eigenvalues of second mode for each simulation for each initial fuel value. The eigenvalue color scale (blue to red) shows the L-2 norm of each eigenvalue’s associated Koopman mode. As in the MTF case, the second mode eigenvalues represent the general dependence of the all-eigenvalue case on the maximum site fuel value.

**Fig 21:**
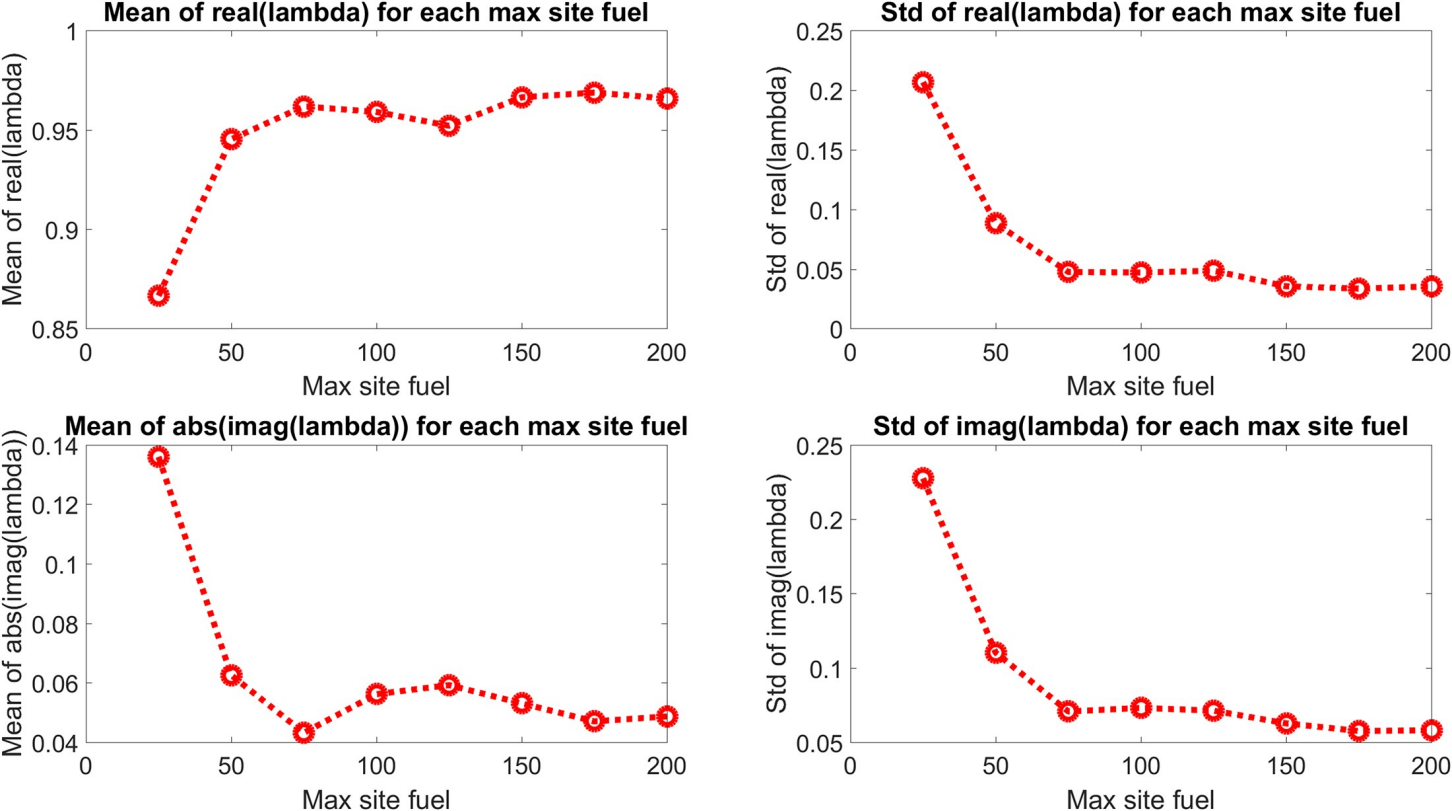
Mean and standard deviation of second mode eigenvalue components. The second mode eigenvalues are seen to approach the unit circle and real axis with increasing values of maximum site fuel value.

Fig 18 shows the eigenvalues for the dominant mode for each site initial fuel value and Fig 19 shows the mean and standard deviation values of the real and imaginary components of those eigenvalues. Figs 20 and 21 show the eigenvalues and their statistics for the second mode for each site initial fuel value. The eigenvalues of the dominant mode are seen to consistently cluster near (and generally inside) the unit circle with small or zero values of the eigenvalue imaginary component. These modes represent the constant (mean) or slowly growing/decaying components of the site fuel level dynamics. Additional information about the site dynamical behavior is apparent in the distribution of the second mode eigenvalues, where the transition from eigenvalues being generally closer to the origin to clustering near the dominant mode eigenvalues as the maximum site fuel value increases represents a decrease in damping or an increase in external driving. This is consistent with the increased occurrences of refueling made possible by the increasing minimum mission life, where the refueling function of the assets serves as a driving force.

**Fig 22:**
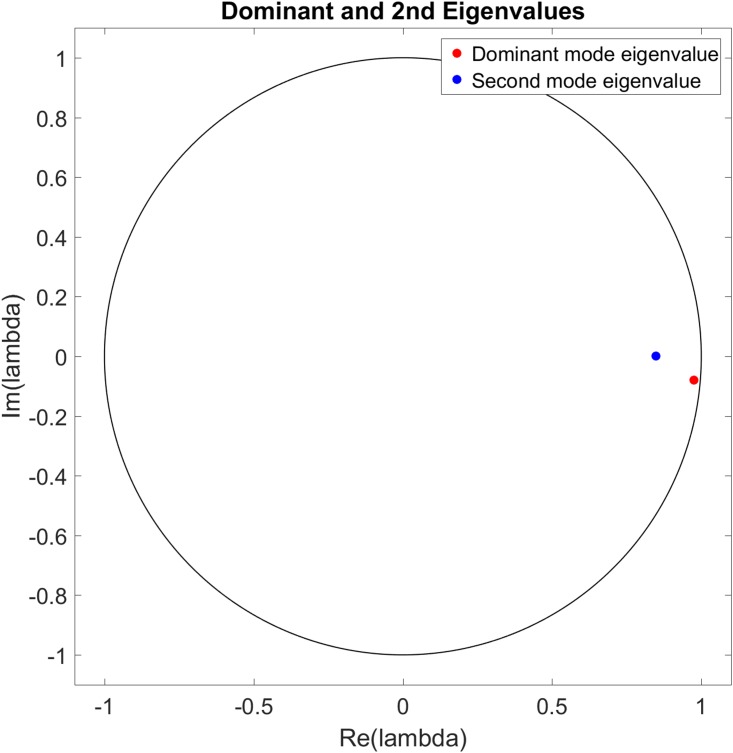
Example eigenvalues of the dominant and second modes from a single simulation for maximum site fuel value of 25 units. The dominant mode eigenvalue is nearly on the unit circle and the real axis, indicating little or no temporal growth/decay or oscillatory behavior, while the second mode eigenvalue is closer to the origin and off of the real axis, indicating long term decay and oscillatory behavior of the associated mode.

**Fig 23:**
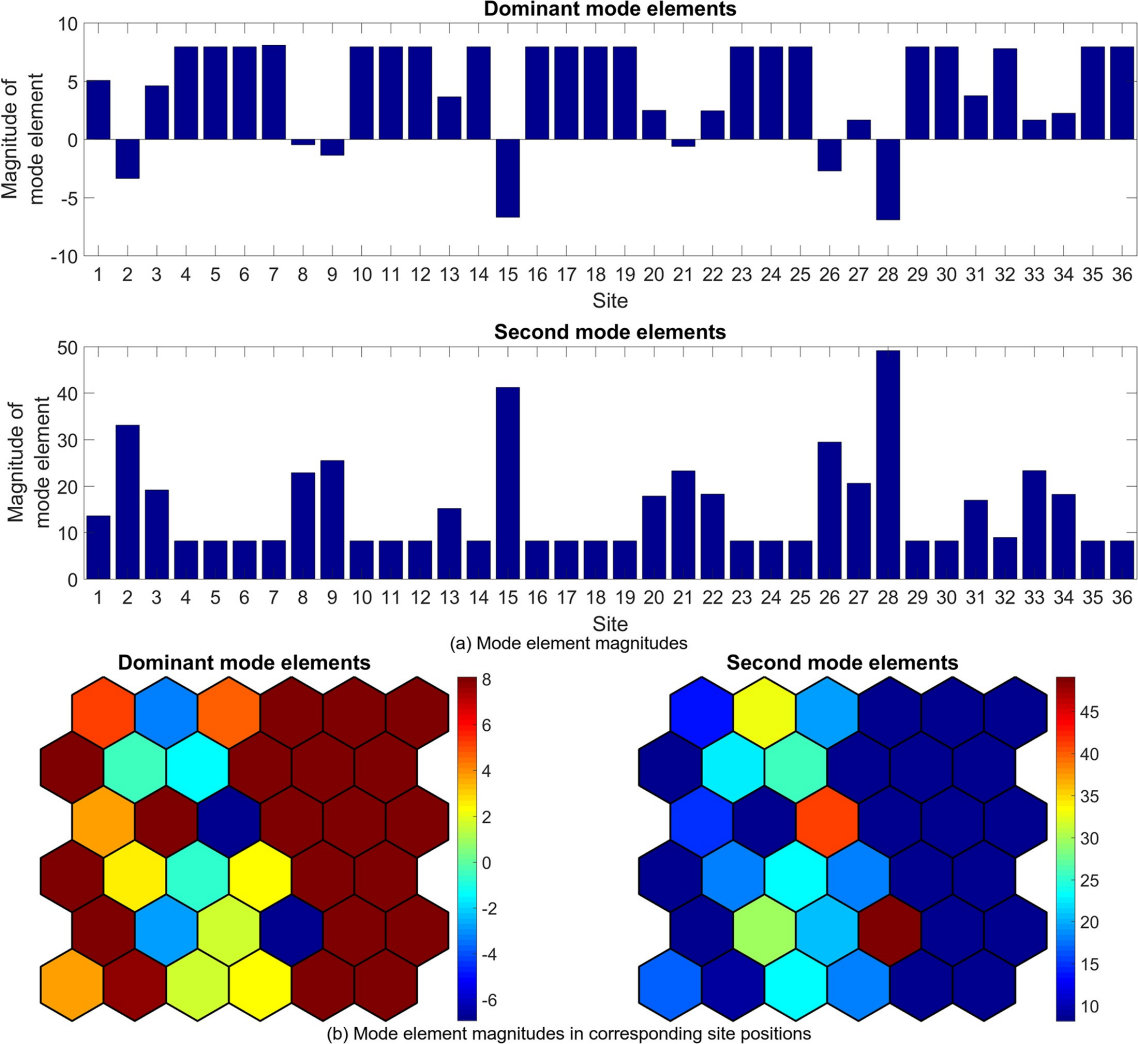
Example mode elements of the dominant and second modes from a single simulation for maximum site fuel value of 25 units, shown in bar graph form (a) and in the spatial positions of the corresponding sites (b). The dominant mode values approximate the mean site fuel values, while the second mode values show the degree to which site fuel values oscillate and/or undergo long-term decay.

**Fig 24:**
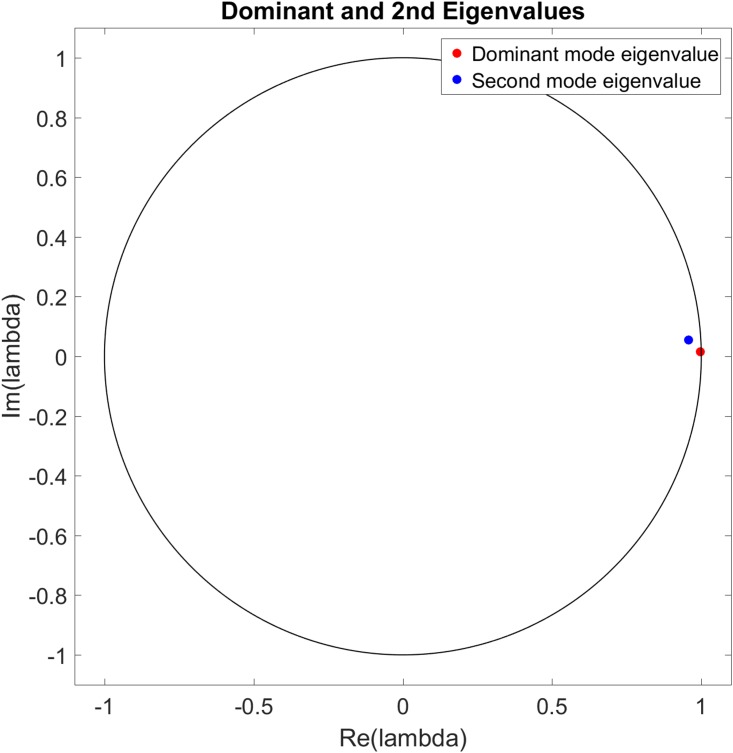
Example eigenvalues of the dominant and second modes from a single simulation for maximum site fuel value of 100 units. The dominant mode eigenvalue is nearly on the unit circle and the real axis, indicating little or no temporal growth/decay or oscillatory behavior, while the second mode eigenvalue is closer to the origin and off of the real axis, indicating long term decay and oscillatory behavior of the associated mode.

**Fig 25:**
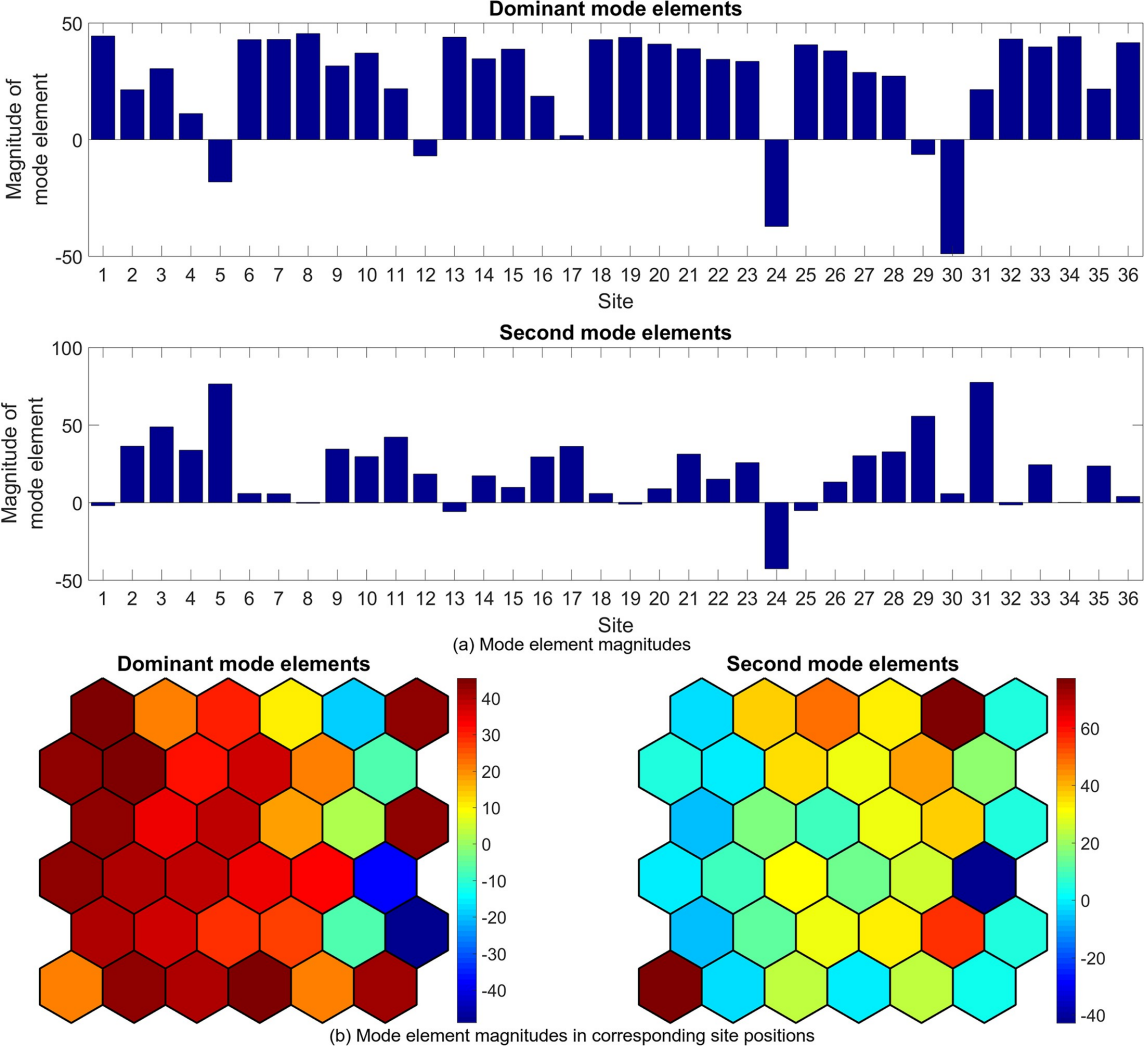
Example mode elements of the dominant and second modes from a single simulation for maximum site fuel value of 100 units, shown in bar graph form (a) and in the spatial positions of the corresponding sites (b). The dominant mode values approximate the mean site fuel values, while the second mode values show the degree to which site fuel values oscillate and/or undergo long-term decay.

**Fig 26:**
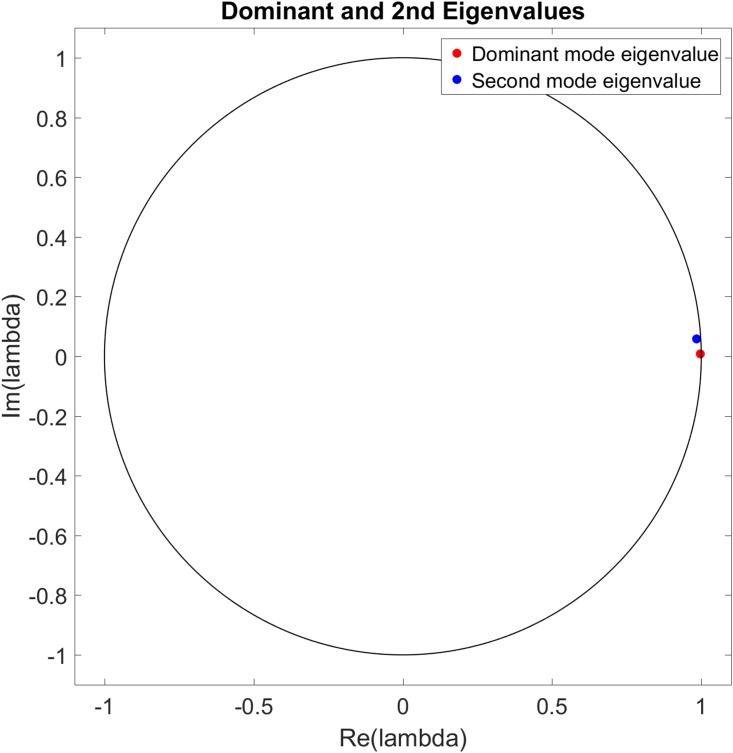
Example eigenvalues of the dominant and second modes from a single simulation for maximum site fuel value of 200 units. The dominant mode eigenvalue is nearly on the unit circle and the real axis, indicating little or no temporal growth/decay or oscillatory behavior, while the second mode eigenvalue is closer to the origin and off of the real axis, indicating long term decay and oscillatory behavior of the associated mode.

**Fig 27:**
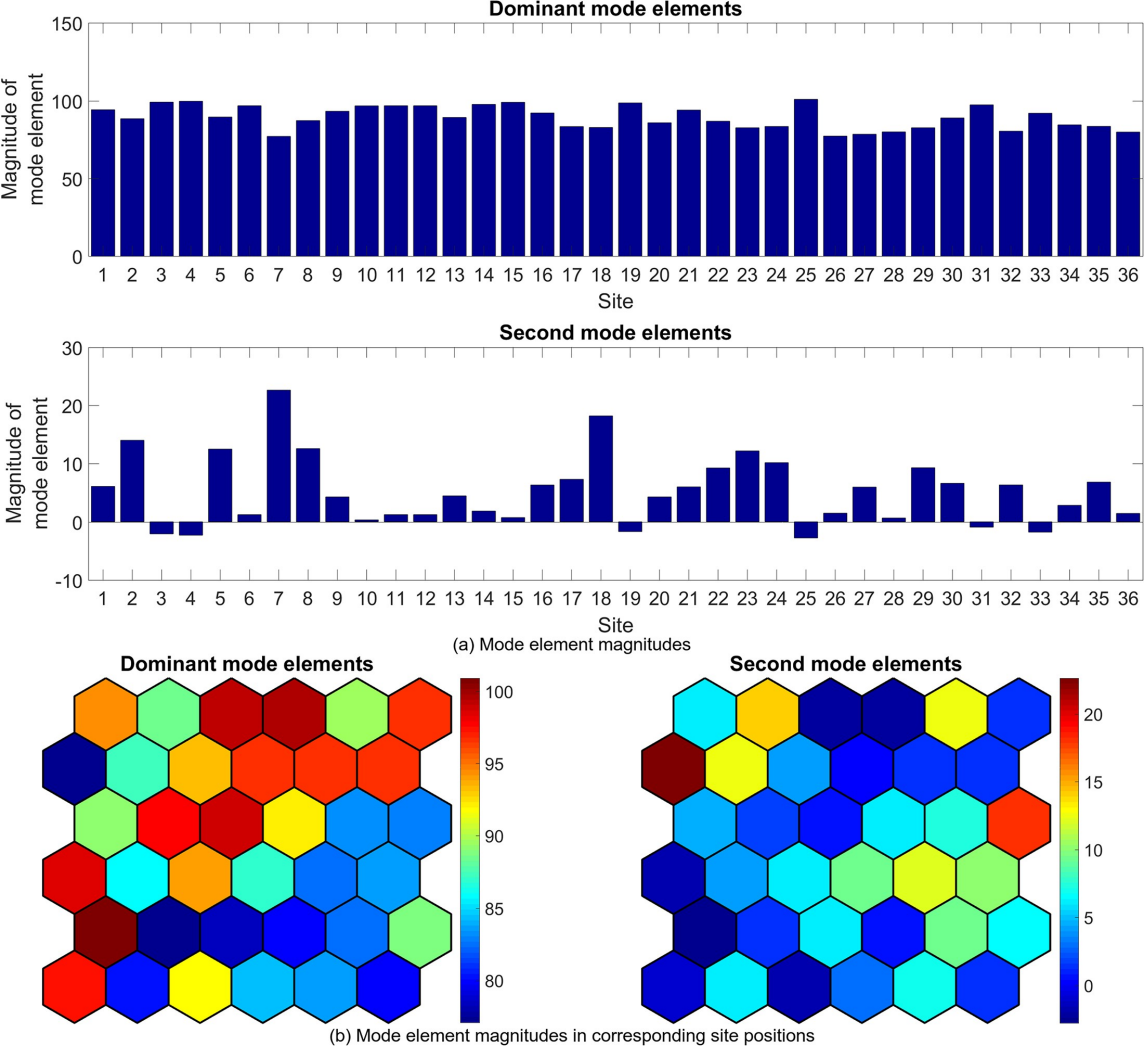
Example mode elements of the dominant and second modes from a single simulation for maximum site fuel value of 200 units, shown in bar graph form (a) and in the spatial positions of the corresponding sites (b). The dominant mode values approximate the mean site fuel values, while the second mode values show the degree to which site fuel values oscillate and/or undergo long-term decay.

Figs 22 to 27 show the dominant and second eigenvalues and corresponding modes (in bar graph and site spatial location form) for single example simulations for maximum site fuel values of 25, 100, and 200 units. Examination of the magnitudes of the mode elements reveals information about the dynamical behavior of each element’s corresponding site. For example, comparison of the time series in Fig 12 with the modes in Fig 23 shows that sites that are refueled one or more times by a significant amount (specifically, sites 1, 2, 3, 8, 9, 13, 15, 20, 21, 22, 26, 27, 28, 31, 32, 33 and 34) have relatively small positive or negative values in the dominant mode and relatively large values in the second mode, while the sites that have monotonically decreasing fuel values (sites 4, 5, 6, 7, 10, 11, 12, 14, 16, 17, 18, 19, 23, 24, 25, 29, 30, 35, and 36) have large positive values in the dominant mode and small positive values in the second mode. Because the dominant mode is a slowly decaying mode (i.e. its eigenvalue is just inside the unit circle) the larger positive mode values of the monotonically decreasing fuel sites correspond to primarily slowly decaying behavior, and the smaller positive or negative values of the refueled sites correspond to more slowly decaying or even growing behavior. Similarly, the second mode is further inside the unit circle and therefore represents faster decaying or more damped behavior, so the large values of the refueled sites represent damping of the applied forcing (i.e., the tendency of the fuel level to continue decreasing after being made to increase). Similar remarks apply to the 100 and 200 maximum site fuel value cases.

DMD decomposes signals into *exponentially* growing or decaying modes. However, in this problem, we have linearly decaying signals with random jumps. A priori, we have little intuition on what the true KMD spectrum is for such signals. As can be seen with the above results, DMD numerically computes a cluster of eigenvalues around 1 in the complex plane for such signals. Given this mismatch in the essential character of the signal (linear) and the approximating functions (exponential), one may wonder about the validity of the computed spectrum. In the next section, we perform a theoretical analysis of the algorithm that justifies the above numerical results. The analysis shows that using complex exponentials to approximate linear signals with random jumps forces repeated roots at 1. The clustering of eigenvalues around 1 is then due to numerical splitting of the repeated roots.

### Approximating linear signals with KMD

In the ship fueling logistics problem, we have signals whose components decay linearly in time with random jumps in the signal. For these signals, we see a cluster of eigenvalues around 1 in the complex plane (see Fig 18). We argue here that this is due to a numerical splitting of repeated eigenvalues at *z* = 1. We show the analytic result for 2 random jumps. The argument can easily extended by induction. In what follows, we will be using notation from the Koopman Mode Decomposition section above.

Let $1_{n}=\left(1,\ldots ,1\right)\in R^{n}$, and let *c* > 0 be the fuel capacity of the sites. Let *t*_1_ < *t*_2_ be positive integers which represent the random times when fuel is dropped at the sites. Let $z_{1},z_{2}\in {\left(R^{+}\right)}^{n}$ be the amount of fuel dropped at the *n* sites at times *t*_1_ and *t*_2_, respectively. The evolution of the fuel at the sites is given by

$${\text{f}}_{0}\;=\;c1_{n}$$ (11)

$${\text{f}}_{k}\;=\;{\text{f}}_{0}-\left(k\Delta t\right)1_{n}+H\left(k-t_{1}\right){\text{z}}_{1}+H\left(k-t_{2}\right){\text{z}}_{2},$$ (12)

where 0 < Δ*t* ≪ *c* is the time step of the simulation, *H*(*t*) is the Heaviside step function (=1 for *t* ≥ 0 and 0 otherwise). We assume that *m* − 1 > *t*_2_.

In this case, **X** = (**f**_0_, …, **f**_*m*−1_) is spanned by

$$\begin{array}{c} B_{3}=\left\{1_{n},z_{1},z_{2}\right\}. \end{array}$$ (13)

The singular value decomposition of **X** can be expanded as

$$\begin{array}{c} X=K\Sigma W^{*}=[K_{3}|K_{n-3}]\left[\begin{array}{cc} S & 0\\ 0 & 0 \end{array}\right]\left[\frac{W_{3}^{*}}{W_{m-3}^{*}}\right] \end{array}$$ (14)

where **S** is a 3 × 3 diagonal matrix, $K_{3}\in R^{n\times 3}$, $K_{n-3}\in R^{n\times n-3}$, $W_{3}\in R^{m\times 3}$, and $W_{m-3}\in R^{m\times m-3}$. The DMD matrix is given by

$$\begin{array}{c} A=K_{3}^{*}X^{\prime }W_{3}S^{-1}. \end{array}$$ (15)

where **X**′ = (**f**_1_, …, **f**_*m*_).

Let us look more closely at the structure of **A** in light of (14). First, we recognize that the data is always spanned by **B**_3_ = {**1**_*n*_, **z**_1_, **z**_3_}, and the span of **B**_3_ is the same as the subspace spanned by **K**_3_. Together, these imply that the image of *U*^Δ*t*^ is orthogonal to span **K**_*n*−3_, and thus, $0=K_{n-3}^{*}U^{\Delta t}X=K_{n-3}^{*}X^{\prime }$. Expanding the definition of **A**:

$$\begin{array}{cc} \text{A} & =\left[\frac{{\text{K}}_{3}^{*}}{{\text{K}}_{n-3}^{*}}\right]{\text{X}}^{\prime }\text{W}\Sigma^{+}\;=\;\left[\frac{{\text{K}}_{3}^{*}}{0}\right]{\text{X}}^{\prime }\text{W}\Sigma^{+}\;=\;\left[\frac{{\text{K}}_{3}^{*}}{0}\right]U^{\Delta t}\text{XW}\Sigma^{+}\\ & =\;\left[\frac{{\text{L}}_{3}^{*}}{0}\right]\;U^{\Delta t}([{\text{K}}_{3}\;|\;{\text{K}}_{n-3}]\text{S}{\text{W}}^{*})\text{W}{\text{S}}^{+}\\ & =\;\left[\begin{array}{cc} {\text{K}}_{3}^{*}U^{\Delta t}{\text{K}}_{3} & {\text{K}}_{3}^{*}U^{\Delta t}{\text{K}}_{n-3}\\ 0 & 0 \end{array}\right]\;\left[\begin{array}{cc} \text{I} & 0\\ 0 & 0 \end{array}\right]\\ & =\;\left[\begin{array}{cc} {\text{K}}_{3}^{*}U^{\Delta t}{\text{K}}_{3} & 0\\ 0 & 0 \end{array}\right]\;≕\;[\begin{array}{cc} {\text{A}}_{3} & 0\\ 0 & 0 \end{array}]. \end{array}$$

Therefore, the spectrum of **A** is

$$\begin{array}{c} \sigma \left(A\right)=\sigma \left(K_{3}^{*}U^{\Delta t}K_{3}\right)\cup \left\{0\right\}=\sigma \left(A_{3}\right)\cup \left\{0\right\}. \end{array}$$ (16)

Thus, we need to compute the spectrum of $A_{3}=K_{3}^{*}U^{\Delta t}K_{3}$ to find *σ*(**A**).

#### Analytic spectrum of A_3_

Note that **A**_3_ is the representation of *U*^Δ*t*^ in the basis **U**_3_. Since the spectrum is basis-invariant, we can rewrite *U*^Δ*t*^ in terms of the basis **B**_3_. To do this, we use the relations *U*^*Δt*^**f**_0_ = **f**_1_, $U^{\Delta t}f_{t_{1}}=f_{t_{1}+1}$, and $U^{\Delta t}f_{t_{2}}=f_{t_{2}+1}$.

- For the first basis element, we have
  $$\begin{array}{c} cU^{\Delta t}1_{n}=U^{\Delta t}f_{0}=f_{1}=c1_{n}-\Delta t1_{n}=\left(c-\Delta t\right)1_{n} \end{array}$$ (17)
  Therefore,
  $$\begin{array}{c} U^{\Delta t}1_{n}=(1-\frac{\Delta t}{c})1_{n}. \end{array}$$ (18)
- For the second basis element, we have
  $$U^{\Delta t}{\text{f}}_{t_{1}}\;=\;U^{\Delta t}\left(c1_{n}-t_{1}\Delta t\right)1_{n}+{\text{z}}_{1})\;=\;\left(c-t_{1}\Delta t\right)U^{\Delta t}1_{n}+U^{\Delta t}{\text{z}}_{1}\;=\;\left(c-t_{1}\Delta t\right)\left(1-\frac{\Delta t}{c}\right)1_{n}+U^{\Delta t}{\text{z}}_{1}.$$ (19)
  Furthermore,
  $$\begin{array}{c} U^{\Delta t}f_{t_{1}}=f_{t_{1}+1}=c1_{n}-\left(t_{1}+1\right)\Delta t1_{n}+z_{1} \end{array}\begin{array}{c} =\left(c-t_{1}\Delta t\right)1_{n}-\Delta t1_{n}+z_{1} \end{array}$$ (20)
  Equating (19) and (20) gives that
  $$\begin{array}{c} U^{\Delta t}z_{1}=-t_{1}\frac{\Delta t^{2}}{c}1_{n}+z_{1}. \end{array}$$ (21)
- For the third basis element, we have
  $$\begin{array}{ll} U^{\Delta t}{\text{f}}_{t_{2}} & =\;U^{\Delta t}\left(c1_{n}-t_{2}\Delta t1_{n}+{\text{z}}_{1}+{\text{z}}_{2}\right)=\left(c-t_{2}\Delta t\right)U^{\Delta t}1_{n}+U^{\Delta t}{\text{z}}_{1}+U^{\Delta t}{\text{z}}_{2}\\ & =\;\left(c-t_{2}\Delta t\right)\left(1-\frac{\Delta t}{c}\right)1_{n}+U^{\Delta t}{\text{z}}_{1}+U^{\Delta t}{\text{z}}_{2}. \end{array}$$ (22)
  Furthermore,
  $$\begin{array}{c} U^{\Delta t}{\text{f}}_{t_{2}}={\text{f}}_{t_{2}+1}=c1_{n}-\left(t_{2}+1\right)\Delta t1_{n}+{\text{z}}_{1} \end{array}\begin{array}{c} +{\text{z}}_{2} \end{array}$$ (23)
  $$\begin{array}{cc} & =\left(c-t_{2}\Delta t\right)1_{n}-\Delta t1_{n}+z_{1}+z_{2}. \end{array}$$ (24)
  Equating the two expressions for $U^{\Delta t}f_{t_{2}}$ gives
  $$\begin{array}{c} U^{\Delta t}z_{2}=-\left(t_{2}-t_{1}\right)\frac{\Delta t^{2}}{c}1_{n}+z_{2}. \end{array}$$ (25)

From (18), (21), and (25), we can write the matrix representation of *U*^Δ*t*^ in the **B**_3_ basis as

$$\begin{array}{c} {\left[U^{\Delta t}\right]}_{B_{3}}=[\begin{array}{ccc} 1-\frac{\Delta t}{c} & -t_{1}\frac{\Delta t^{2}}{c} & -\left(t_{2}-t_{1}\right)\frac{\Delta t^{2}}{c}\\ 0 & 1 & 0\\ 0 & 0 & 1 \end{array}]. \end{array}$$ (26)

Since this is an upper triangular matrix,

$$\begin{array}{c} \sigma \left({\left[U^{\Delta t}\right]}_{B_{3}}\right)=\sigma \left(A_{3}\right)=\{(1-\frac{\Delta t}{c}),1,1\}. \end{array}$$ (27)

Finally, the total DMD spectrum is

$$\begin{array}{c} \sigma \left(A\right)=\{1,1,(1-\frac{\Delta t}{c}),0,\ldots ,0\}. \end{array}$$ (28)

For example, if Δ*t* = 1 and the fuel capacity of the sites is *c* = 100, then the spectrum is

$$\begin{array}{c} \sigma \left(A\right)=\{1,1,0.99,0,\ldots ,0\}. \end{array}$$ (29)

While this is the analytic spectrum, numerical errors due to floating point precision will give slightly different answers, as will be seen below.

#### Numerical example: DMD spectrum for linear decay

**Fig 28:**
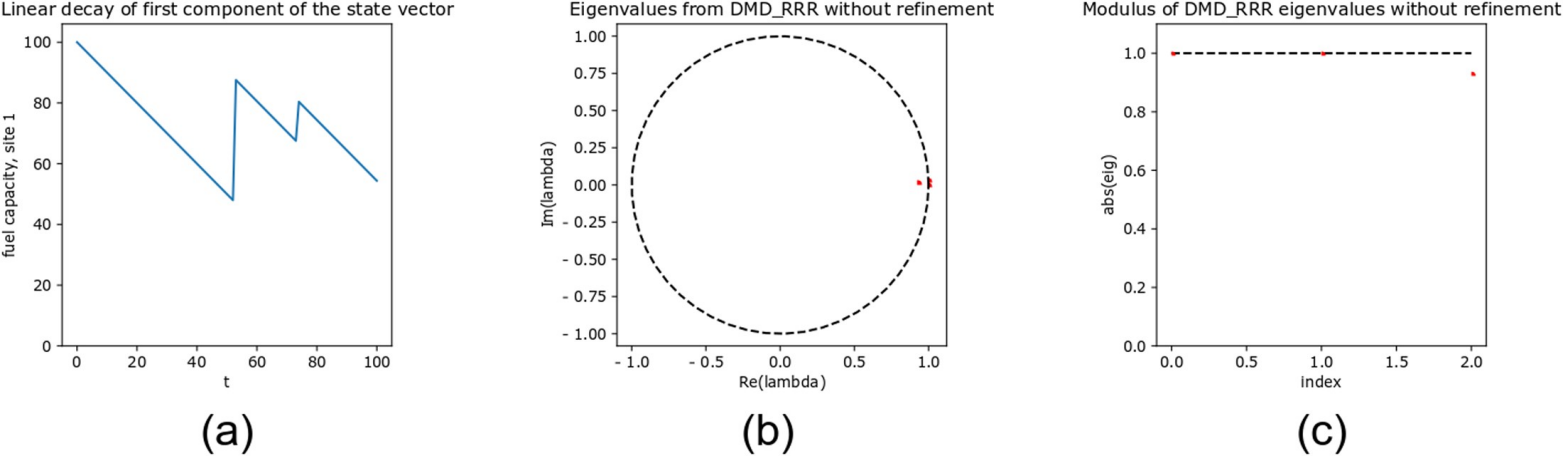
(a) Linear decay with random jumps. (b) DMD_RRR spectrum corresponding to (30). (c) Modulus of the spectrum.

Here, we show that, although the analytically derived values for the DMD spectrum is given by (28), the numerical computation gives slightly different values. For this example, we choose *n* = 50, *m* = 2*n* + 1, Δ*t* = 1, and *c* = 100. The left panel of Fig 28 shows the first component of ${\left\{f_{k}\right\}}_{k=0}^{m-1}$, the middle panel the spectrum as computed by DMD, and the right panel shows the modulus of the spectrum. The numerical values of the spectrum of **A**_3_ computed using DMD_RRR are

$$\begin{array}{c} \begin{array}{cc} \sigma \left(A_{3}\right)=\{ & 0.9918346047900112+0.016040738466815568j,\\ & 0.9918346047900112-0.016040738466815568j,\\ & 0.9227556556053357+0j\} \end{array} \end{array}$$ (30)

## Discussion and conclusions

The application of KMA to the two ABM systems described here is intended to demonstrate the utility of KMA in revealing information about the present and future behavior of the system that is not apparent simply from examination of the observables of the system. It is not intended as an optimization or modeling technique by itself, and the example ABM systems described in this paper were chosen for their dynamical features rather than their fidelity or relevance to real-world SCM or health care systems. Nonetheless, it is instructive to examine existing applications of ABM to optimization problems in SCM and health care systems, and to consider the possible contributions of KMA to these types of models.

There exists an extensive literature on applications of ABM to optimization problems (see Barbati et al for a recent literature review [26]), including applications of ABM-based simulation-optimization to problems in SCM and health care. KMA, while not an optimization technique itself, can be applied to optimization problems in various ways. For example, Oremland and Laubenbacher [27] describe a model reduction technique for ABM optimization, which could be aided by KMA by identifying the most significant temporal and spatial behaviors of the full model to produce and validate an appropriate reduced order model.

The SCM literature includes a large number of applications of ABM. The reader is referred to, e.g. Arvitrida’s [28] review of ABM in SCM, specifically in a collaboration context, and to the extensive literature review of Fuller et al [29]. The common problem inherent to the application of ABM to SCM and other similarly complex systems is how to determine, in a general sense, the model parameters required to accurately reproduce existing systems and/or to produce an optimal system. This problem can be described as the determination, explicitly or implicitly, of a cost or reward function, where for a given state of the system the function outputs the expected optimal decision or action. The computational cost, for a given algorithm, of solving such an optimization problem depends on the state space over which the optimization process occurs. KMA can potentially improve the effectiveness of solving such optimization problems, where representation of the system state by decomposition of the past history of the system observables into Koopman eigenvalues and modes describing the temporal and spatial dependencies of the system dynamics simplifies the optimization process. As a specific example, Fuller et al [29] describe an ABM using learning agents where an efficient model of the oil industry value chain is produced without a priori or expert knowledge of optimal policies. In their approach, the agents learn an appropriate reward function early in the simulation then apply that learned reward function to choose actions in the rest of the simulation. The optimality of the model is therefore based on how well the learned reward function for a given state of the system matches the true reward function, which in turn is based on how effectively the learning algorithm uses the state space information of the system. The effectiveness of the learning algorithm can potentially be improved by use of the Koopman eigenvalues and mode as inputs to the learning algorithm, in addition to the current system state. Such an approach includes the greater information about the system response by condensing the system dynamics into single values, rather than requiring the details of the past system behavior to be determined from the entire set of previous states of the system.

There also exists literature on applications of ABM or discrete event simulation (DSE) based optimization to health care resource planning, which could potentially benefit from KMA-derived insights. Cabrera et al [30], Weng et al [31], Wong et al [32], and Zeinali et al [33] describe ABM or DES based approaches to create decision support systems for hospital staffing and resource allocation to reduce patient wait times or optimize resource usage. Common features of such systems are the stochastic nature of the arrival times and numbers of incoming patients, and the varied duration of the patients’ stay in the hospital. In our analysis of the MTF system, applying KMA to the time series of patient numbers can provide actionable information on when the available patient capacity of a facility is likely to be exceeded, thus giving warning of the need to re-allocate resources to meet patient care demands. Yousefi et al [34] describe an emergency department resource planning model wherein a metamodel is constructed based on an ABM simulation, and genetic algorithms and neural network based methods are used to train the metamodel. Such an approach could benefit from application of KMA to both the metamodel construction task, where a sensitivity analysis of the time series information could inform the essential features of the ABM simulation to include in the metamodel, and also the Koopman eigenvalues and modes can be used as features to train upon which to train the neural networks, where those KMA derived features each have a simple time dependence and thus generally simplify the task of training a neural network to recognize the essential system dynamics [35], [36].

For both the MTF and SF logistics systems, analysis of the Koopman eigenvalues is seen to provide useful information about the state of the system. In both cases, analysis of the Koopman eigenvalues shows the change in system dynamics from one regime to another as a control parameter is varied, specifically from a strongly damped, fluctuation-dissipation regime to a weakly or non-damped oscillatory regime. In the MTF system, this change in dynamics is due to the increasing number of incoming casualties overwhelming the MTFs and causing the system to shift to a dangerous, high death rate state. For the ship fueling logistics system, increasing site fuel capacity leads to a positive outcome as the refueling assets are more effective as sites can last longer between being refueled.

It is easy to see that, if a signal is a combination of an exponential signals, DMD will compute the correct spectrum. However, for linear signals with random jumps—such as in the ship fueling logistics problem—it is not a priori obvious that DMD will correctly compute the spectrum or, indeed, if the spectrum is meaningful. We took up the justification of our numerical results for the ship fueling logistics problem with a theoretical analysis of the DMD algorithm applied to linearly decaying signals with random jumps. Such signals force the DMD spectrum to have repeated eigenvalues at *z* = 1 in the complex plane, with the number of repeated eigenvalues equal to the number of random jumps in the signal. Numerical inaccuracies due to floating point arithmetic split these repeated eigenvalues resulting the cluster of eigenvalues around 1 that were seen in our results.

Our results indicate that tracking of bifurcations in complex logistic systems is possible using Koopman Mode Analysis. We have studied two different logistics systems here, with two different types of dynamics and concluded that indicators of performance can be built based on selected Koopman Modes. It would be of great interest to extend these types of studies using only observational data from logistics systems.

## Data management

All datasets generated by our models are housed in a Zenodo public repository and can be found at: http://doi.org/10.5281/zenodo.2567599. The datasets possess their own DOI number and can be cited as [37].
